# Circular RNA circLIMK1-005 promotes the progression of lung adenocarcinoma by interacting with RPA1 protein to activate CDK4 signaling

**DOI:** 10.1038/s41420-025-02565-y

**Published:** 2025-07-01

**Authors:** Xia Yang, Lu Liu, Zhongjian Yu, Yuanlin Chen, Shiting Xu, Meiyuan Liu, Meng Wang, Huili Guo, Zhiwu Zhang, Bingjie Shan, Silin Cai, Mengting Pan, Jiangyu Zhang, Fengpin Wang, Yanfang Zheng

**Affiliations:** Guangzhou Institute of Cancer Research, the Affiliated Cancer Hospital, Guangzhou Medical University, Guangzhou, China

**Keywords:** Non-small-cell lung cancer, Non-small-cell lung cancer, Tumour biomarkers

## Abstract

Lung adenocarcinoma (LUAD) is the leading cause of cancer death worldwide. Circular RNAs (circRNAs) have emerged as potential key players in the onset and progression of various cancers. However, the specific roles and mechanisms of circRNAs in LUAD remain largely unexplored. Here, we aimed to elucidate the role of a particular novel circRNA, circLIMK1-005 (hsa_circ_0002690), in the pathogenesis of LUAD. Our study revealed that circLIMK1-005 was upregulated in LUAD and correlated with poor patient prognosis. Functionally, circLIMK1-005 significantly promoted LUAD cell proliferation and metastasis. Mechanistically, circLIMK1-005 elevated the expression of Cyclin D1 and CDK4 proteins, thereby activating CDK4 signaling. We further demonstrated that circLIMK1-005 promoted LUAD progression by binding with RPA1 protein and activating the CDK4 pathway. In vivo experiments corroborated these findings, confirming that the circLIMK1-005/RPA1/CDK4 axis contributed to LUAD progression and was associated with poor clinical outcomes. Our study revealed a novel mechanism of the circLIMK1-005/RPA1/CDK4 axis in LUAD progression, and highlighted that targeting circLIMK1-005 could represent a potential therapeutic strategy for patients with LUAD.

**Figure:**
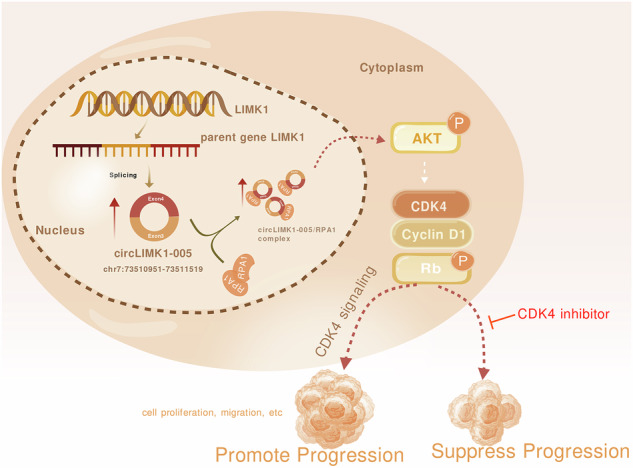
Schematic diagram of hypothesis involved in the circLIMK1-005/RPA1/CDK4 axis in LUAD progression. Figure was created with BioGDP.com.

Schematic diagram of hypothesis involved in the circLIMK1-005/RPA1/CDK4 axis in LUAD progression. Figure was created with BioGDP.com.

## Introduction

According to global cancer statistics, lung cancer is the leading cause of cancer-related morbidity and mortality in China [1]. Non-small cell lung cancer (NSCLC) accounts for approximately 85% of all lung cancer cases [2], with adenocarcinoma being the most common pathological type of NSCLC [3]. Despite advancements in treatments such as curative resection, targeted therapies, chemotherapy, and immunotherapy, the overall prognosis for LUAD remains poor. This is largely because most patients are diagnosed at advanced stages with distant metastases, given the often-asymptomatic nature of early-stage LUAD [4]. Consequently, further research into the mechanisms underlying LUAD oncogenesis and progression is of critical clinical importance and could facilitate the development of novel therapeutic approaches.

Uncontrolled cell division is a well-established cancer hallmark and is a major therapeutic target [5]. Cyclin-dependent kinases 4 and 6 (CDK4/6) are pivotal mediators in the cellular transition to the S phase of the cell cycle. Persistent activation of the Cyclin D1–CDK4/6 axis is a key driver in the tumorigenesis of various cancers [6, 7], including lung cancer [8–10]. Pharmacological inhibitors of CDK4/6 have become the standard of care in advanced hormone receptor-positive breast cancer [7, 11] and have shown promising results in clinical trials across multiple cancer types [11], including lung cancer [12]. Therefore, exploring the broader applicability of CDK4/6 inhibitors in various cancers remains an area of unmet clinical need.

Replication Protein A (RPA) is a heterotrimeric complex that binds to single-stranded DNA (ssDNA), consisting of three subunits: RPA1 (70 kDa), RPA2 (32 kDa), and RPA3 (14 kDa) [13]. It plays crucial roles in DNA replication [14], recombination, DNA damage repair/response [14–16], and transcription [17–20]. Many RPA-ssDNA interacting proteins have been characterized, and the RPA-ssDNA complex serves as a central hub that coordinates a myriad of interacting proteins, thereby maintaining genome stability [13]. Additionally, RPA-ssDNA recruits subunits of DNA polymerases and DNA-repair factors, facilitating DNA replication [14], inducing cell cycle arrest [16], activating DNA damage response and maintaining genome stability [13, 15]. Furthermore, the complex can regulate transcription by recruiting RNA Polymerase II and other transcription factors [17–20]. Thus, RPA complexes play a critical role in both genome stability and transcription regulation. RPA1, the largest subunit of the RPA complex, possesses four DNA-binding domains (DBDs) [13] and is particularly important in tumorigenesis [21, 22]. Given that genetic mutations are a hallmark of LUAD and chromosomal stability serves as a prognostic indicator [8], RPA1 is postulated to play a significant role in LUAD pathogenesis. While some studies have touched upon the regulatory involvement of RPA1 in LUAD [19, 21, 23, 24], its pathological functions and mechanisms within this context remain inadequately explored.

Circular RNAs have garnered attention as a prominent class of primarily non-coding RNA molecules, many of which are implicated in cancer pathogenesis and progression through a variety of mechanisms [25, 26]. A significant function of circRNAs is their ability to serve as molecular sponges for microRNAs (miRNAs), thereby competitively binding to miRNAs and modulating downstream gene expression [27]. Furthermore, circRNAs can engage as interaction partners for proteins, facilitating protein-protein and protein-DNA interactions, as well as protein transport [28, 29]. Recent comprehensive studies by Wang et al. have delineated the landscape of differentially expressed circRNAs in solid tumors [30, 31]. These investigations have disclosed that dysregulated circRNAs either exhibit cancer-specific expression patterns or share common expression signatures across various cancer types. Such aberrant expression could be attributed to alterations in host genes and/or RNA-binding proteins involved in cancer. Over 20 circRNAs have been implicated in the progression of LUAD, underscoring their potential as therapeutic targets [32, 33]. However, the functional roles and underlying mechanisms of circRNAs in LUAD remain largely unexplored. Thus, there is an unmet need to identify and investigate novel circRNAs to elucidate their contributions to LUAD progression.

In a previous study, we performed sequencing analyses on both cancerous and adjacent non-cancerous tissues from LUAD patients to explore the role of circHMGA2 in the progression of LUAD [34]. In the present study, our focus shifted to the functional characterization and mechanistic investigation of a novel circular RNA, circLIMK1-005 (hsa_circ_0002690), in the context of LUAD. Our results revealed that circLIMK1-005 was significantly upregulated in LUAD tissues and was positively associated with poor clinical outcomes. We found that circLIMK1-005 profoundly influenced the proliferation and migratory capacities of LUAD cells. On a mechanical level, our data elaborated that circLIMK1-005 elevated the expression of Cyclin D1 and CDK4 proteins, thereby activating the CDK4 signaling pathway. Furthermore, our analyses demonstrated that circLIMK1-005 interacted with RPA1 protein to activate CDK4 signaling, thereby contributing to LUAD progression. In vivo experiments corroborated these findings, establishing a critical role for the circLIMK1-005/RPA1/CDK4 axis in LUAD pathology. Taken together, our study suggested that circLIMK1-005 represented a promising therapeutic target for managing LUAD patients.

## Results

### circLIMK1-005 was highly expressed in LUAD and positively correlated with poor prognosis

**Fig. 1:**
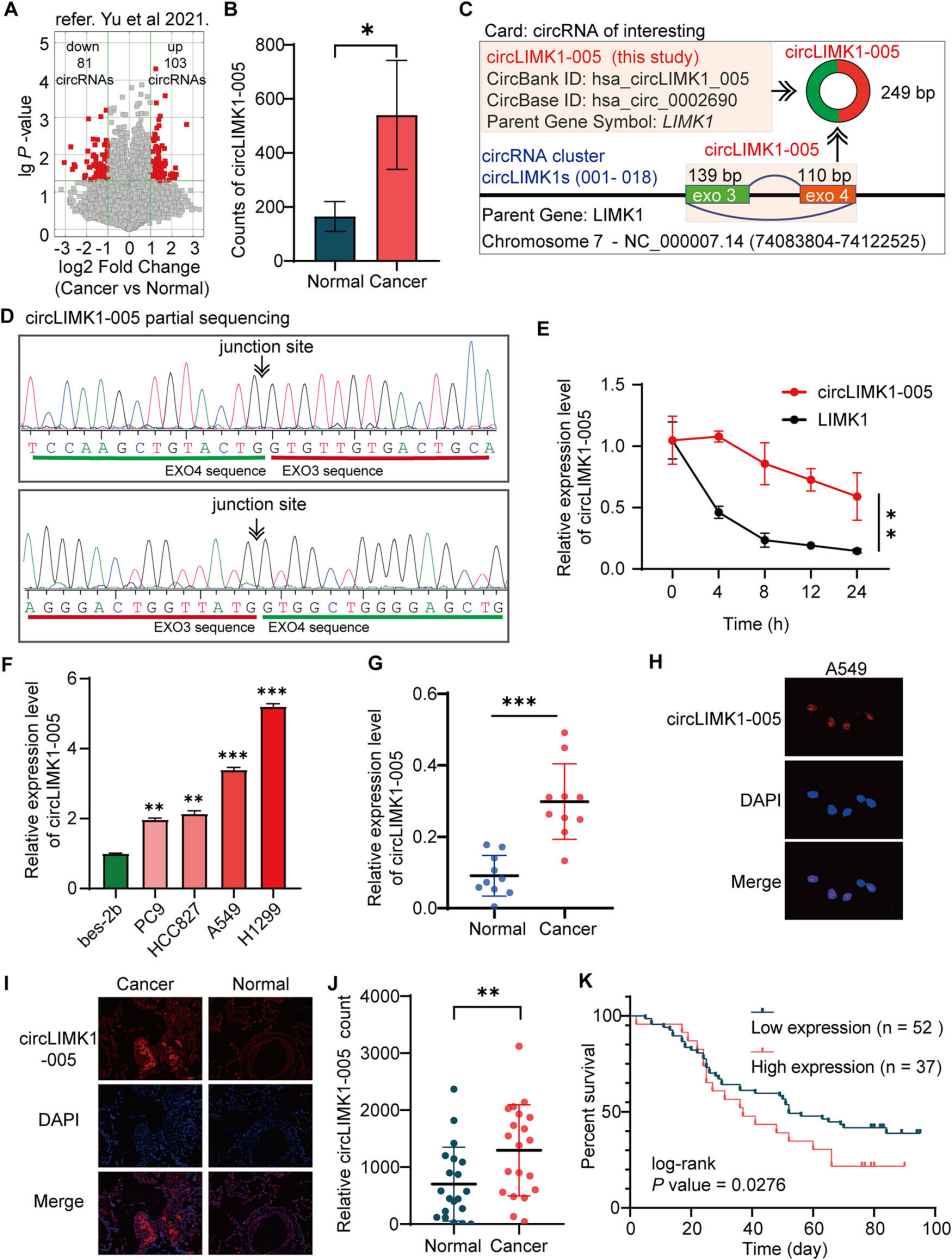
circLIMK1-005 was highly expressed in LUAD and positively correlated with LUAD prognosis. **A** Volcano map of differentially expressed circRNA in LUAD patients (refer to Yu et al. [34]). **B** Relative counts of circLIMK1-005 in LUAD and adjacent normal tissues. **C** Information of circLIMK1-005. **D** Sequencing peak of junction site from circLIMK1-005. **E** A549 cells were treated with actinomycin D, followed by RT-qPCR assay, to verify the stability of circLIMK1-005. **F** RT-qPCR was implemented to investigate the relative expression of circLIMK1-005 in human LUAD cell lines and human lung epithelial cell lines. **G** The relative expression of circLIMK1-005 in LUAD and adjacent normal tissues using RT-qPCR. **H**, **I** FISH with circLIMK1-005 probe (red) and DAPI (green) in A549 cells and tissues of LUAD patients. **J** The relative highly expressed circLIMK1-005 cells from tissues of LUAD patients using FISH. **K** Kaplan–Meier survival analysis of circLIMK1-005 in patients with LUAD.

In our previous transcriptomic analysis of tissues from LUAD patients [34] (Fig. 1A), we identified a novel circular RNA, circLIMK1-005, that was significantly upregulated in LUAD tissues (Fig. 1B). According to the circBase database (http://www.circbase.org/), circLIMK1-005 (has_circ_0002690) is located on human chromosome 7 (chr7: 73510951-73511519) and is generated through reverse splicing from exon 3 to exon 4 of the parent gene LIMK1 (Fig. 1C). According to the circBank database (http://www.circbank.cn/), the LIMK1 gene contributes to 18 circRNAs, numbered as circLIMK1-001 to circLIMK1-018 (Fig. 1C; Supplementary Table S1). Notably, our data indicated that both circLIMK1-005 and circLIMK1-014 were overexpressed in LUAD tissues (Supplementary Fig. S1). Previous studies have implicated the parent gene LIMK1 in poor prognosis and increased immune infiltration in lung cancer [35–37], suggesting a pivotal role for LIMK1 and its associated circRNAs in LUAD progression. Concurrently, another research team has explored the role of circLIMK1-014 in LUAD [38]. Collectively, these investigations suggested that circLIMK1s could serve as promising therapeutic targets in LUAD. In the present study, we focused on elucidating the specific role of circLIMK1-005 in LUAD.

To corroborate the circular nature of circLIMK1-005, we carried out a series of experiments in A549 cells. We initially performed RT-PCR to amplify the circLIMK1-005 product, followed by Sanger sequencing. This enabled us to verify the back-splicing junction between exon 3 and 4 of the parent LIMK1 gene (Fig. 1D; Supplementary Fig. S2). Additionally, actinomycin D experiments for further validating the circular structure of circLIMK1 revealed that circLIMK1-005 demonstrated enhanced stability in comparison to its linear LIMK1 mRNA counterpart (Fig. 1E**;** the full length of circLIMK1-005, Supplementary Table S2).

The expression of circLIMK1-005 in LUAD cell lines and tissues assessed utilizing RT-qPCR demonstrated that circLIMK1-005 expression was upregulated in LUAD cells compared to a human lung epithelial cell (Fig. 1F). Similarly, circLIMK1-005 was highly expressed in LUAD tissues compared to adjacent normal tissues (Fig. 1G). FISH assay on A549 cells and an additional 20 pairs of tumor and adjacent normal tissues revealed that circLIMK1-005 was predominantly localized in the nucleus (Fig. 1H, I). Moreover, an elevated number of cells expressing high levels of circLIMK1-005 was observed in LUAD tissues (Fig. 1J). To evaluate the clinical relevance of circLIMK1-005 expression, we conducted Kaplan–Meier survival analysis on a cohort of 89 LUAD patients, whose tissues were subjected to FISH assays. The analysis established a significant association between elevated circLIMK1-005 expression and poor prognosis in LUAD patients (Fig. 1K). Taken together, these findings revealed that circLIMK1-005 was highly expressed in LUAD and positively correlated with poor prognosis.

### Highly expressed circLIMK1-005 promoted the proliferation and metastasis of LUAD cells

**Fig. 2:**
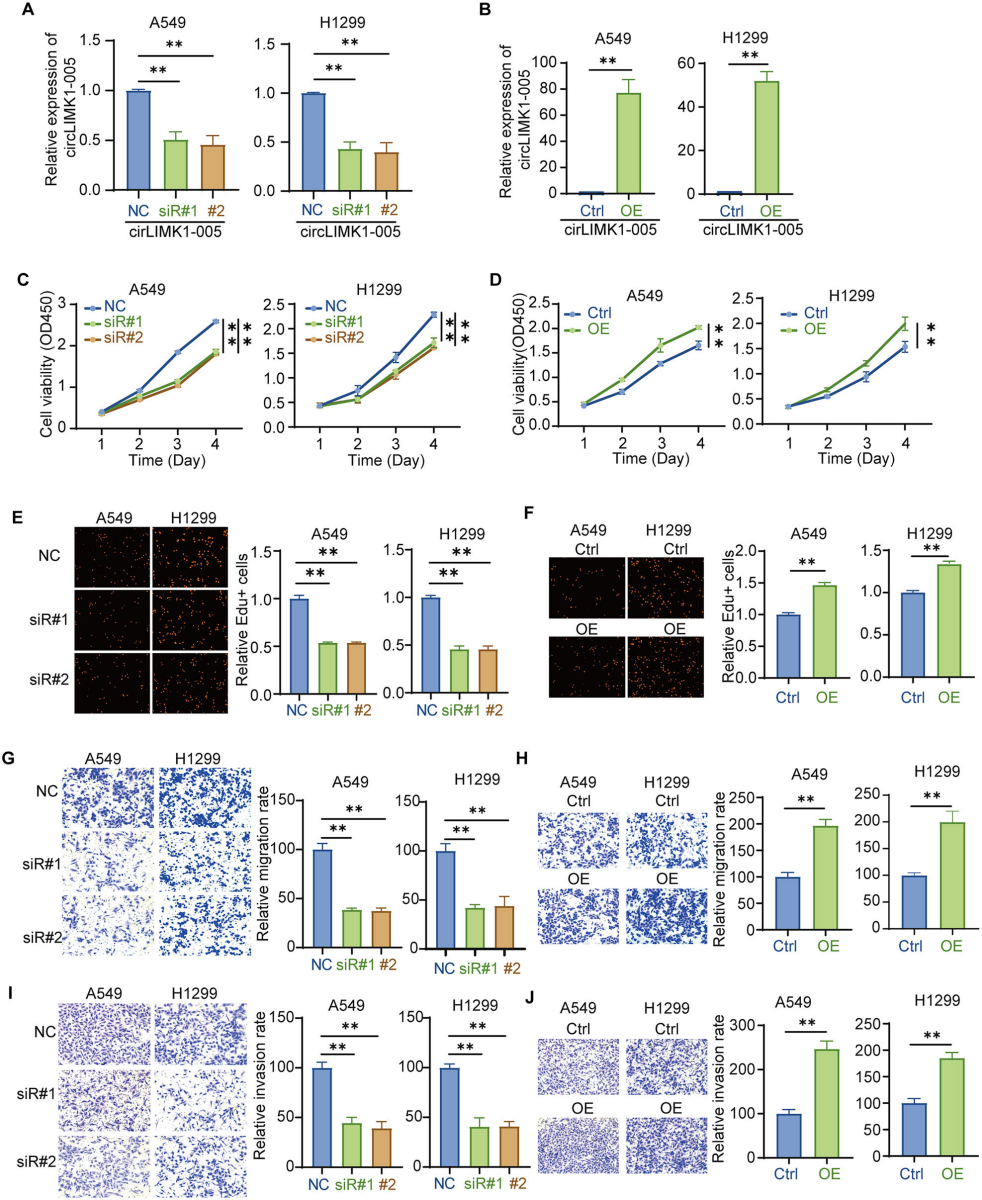
Highly expressed circLIMK1-005 promoted the proliferation and metastasis of LUAD cells. **A**, **B** RT-qPCR of circLIMK1-005 in A549 and H1299 cells to verify the efficiency of circLIMK1-005 knockdown and overexpression. **C**, **D** CCK8 assay was used to investigate cell viability affected by circLIMK1-005 knockdown or overexpression in A549 and H1299 cells. **E**, **F** EdU assay was applied to detect the DNA replication rate regulated by circLIMK1-005 knockdown or overexpression in A549 and H1299 cells. **G**–**J** Transwell assay was performed to assess the ability of migration and invasion regulated by circLIMK1-005 knockdown or overexpression in A549 and H1299 cells. *P value < 0.05, **P value < 0.01.

To elucidate the functional role of circLIMK1-005, we performed experiments in A549 and H1299 cell lines. We generated both circLIMK1-005 knockdown (circLIMK1-005 siRNA) and overexpression (circLIMK1-005 OE) cell models. The efficacy of the knockdown and overexpression was confirmed using RT-qPCR (Fig. 2A, B). The role of circLIMK1-005 in proliferation was explored by CCK8 and EdU assays. The CCK8 assay demonstrated that circLIMK1-005 knockdown significantly attenuated cell viability in A549 and H1299 cells (Fig. 2C), while ectopic overexpression of circLIMK1-005 enhanced cell viability (Fig. 2D). Similarly, the EdU assay indicated that the knockdown of circLIMK1-005 significantly impaired DNA synthesis in A549 and H1299 cells (Fig. 2E; Supplementary Fig. S3), whereas its overexpression promoted DNA synthesis (Fig. 2F; Supplementary Fig. S3). Collectively, circLIMK1-005 played a pivotal role in promoting the proliferation of LUAD cells.

Metastasis is the predominant cause of cancer-related mortality [39]. In vitro cellular research frequently employs cell migration and invasion assays to investigate the metastatic potential of tumor cells. Migration reflects the ability of tumor cells to move, while invasion assesses their capacity to breach the extracellular matrix and invade surrounding tissues. To explore the role of circLIMK1-005 in metastasis in LUAD, we employed transwell-based migration and invasion assays to assess the metastatic potential of this circRNA in A549 and H1299 cells. Our data revealed that the knockdown of circLIMK1-005 led to a significant reduction in the metastatic capabilities of A549 and H1299 cells (Fig. 2G, I). Conversely, the overexpression of circLIMK1-005 in cells resulted in the opposite effect (Fig. 2H, J). In summary, these findings established that elevated levels of circLIMK1-005 contributed to both the enhanced proliferation and increased metastatic activity of LUAD cells, highlighting that circLIMK1-005 may act as a facilitator in the progression of LUAD.

### CircLIMK1-005 promoted LUAD progression via enhancing CDK4 signaling

**Fig. 3:**
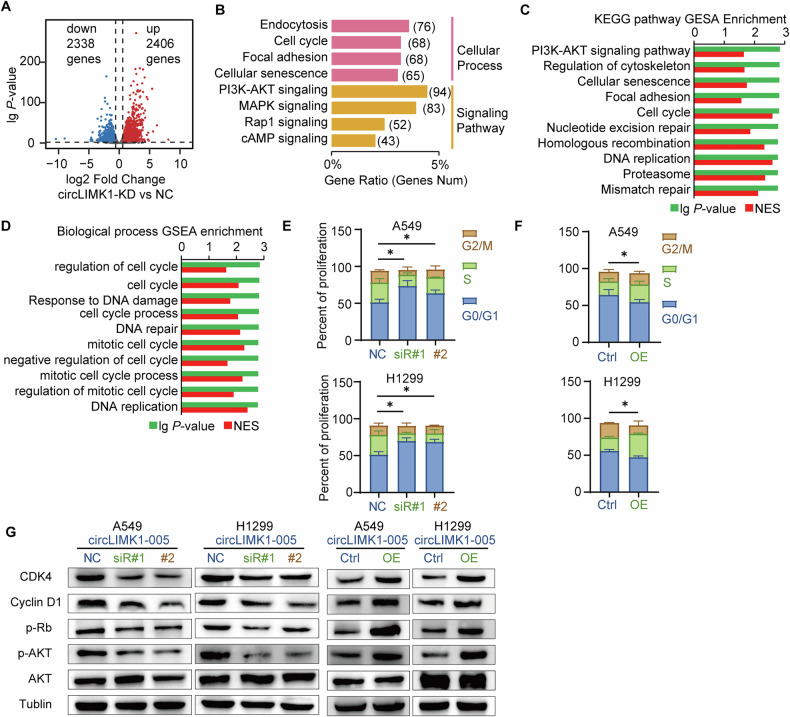
CircLIMK1-005 accelerated LUAD progression via enhancing CDK4 signaling. **A** Volcano map of differentially expressed genes regulated by circLIMK1-005 using transcriptome sequencing. **B** The KEGG analysis of differentially expressed genes. **C** The GSEA enrichment analysis of the KEGG pathway. **D** The GSEA enrichment analysis of GO biological process. **E**, **F** Cell cycle assay using EdU-staining detected by flow cytometry in circLIMK1-005 knockdown or overexpression cells. **G** Western blot with Cyclin D1, CDK4, RB, and AKT antibodies in circLIMK1-005 knockdown or overexpression cells. *P value < 0.05, **P value < 0.01.

To decipher the regulatory mechanisms underpinning the actions of circLIMK1-005 in A549 and H1299 cells, we initially performed transcriptome sequencing on A549 cells subjected to circLIMK1-005 knockdown (KD) versus normal controls (NC). Transcriptomic profiling revealed differential expression of 4744 mRNAs, as visualized in a volcano plot (Fold change >2; *P*-value < 0.05) (Fig. 3A). Subsequent KEGG pathway analysis of these differentially expressed genes pinpointed endocytosis, cell cycle, focal adhesion, and cellular senescence as the four most impacted cellular pathways. Additionally, significant modulation was observed in the PI3K-AKT, MAPK, RAP1, and cAMP signaling pathways (Fig. 3B). Intriguingly, GSEA further highlighted notable effects on cell cycle regulation, DNA damage response signaling, and AKT signaling (Fig. 3C, D). These findings suggested that circLIMK1-005 primarily regulated cell cycle and DNA damage response. For validation, an EdU-staining cell cycle assay was conducted using flow cytometry. The results demonstrated that circLIMK1-005 knockdown led to a notable arrest of cells in the G0/G1 phase (Fig. 3E), while the opposite effect was observed in cells overexpressing circLIMK1-005 (Fig. 3F). These results demonstrated that circLIMK1-005 regulated the transition of the cell cycle from the G0/G1 phase to the S phase in LUAD cells.

The Cyclin D1/CDK4 complex is a pivotal regulator that governs the cellular transition from the G0/G1 phase to the S phase of the cell cycle [6, 7]. Given this established role, we hypothesized that circLIMK1-005 could facilitate the progression of LUAD by modulating CDK4 signaling. For validation, a western blot was implemented in A549 and H1299 cells with Cyclin D1, CDK4, RB and AKT antibodies. Our data revealed that circLIMK1-005 knockdown resulted in decreased levels of Cyclin D1, CDK4, phospho-RB, and phospho-AKT proteins, while overexpression led to the opposite effect (Fig. 3G). These results supported the notion that circLIMK1-005 acted as an activator of CDK4 signaling. Collectively, our results suggested that circLIMK1-005 facilitated the proliferation and metastasis of LUAD cells by upregulating Cyclin D1 and CDK4 protein levels, thereby activating CDK4 signaling and contributing to LUAD progression.

### CircLIMK1 interacted with RPA1 and increased the protein level

**Fig. 4:**
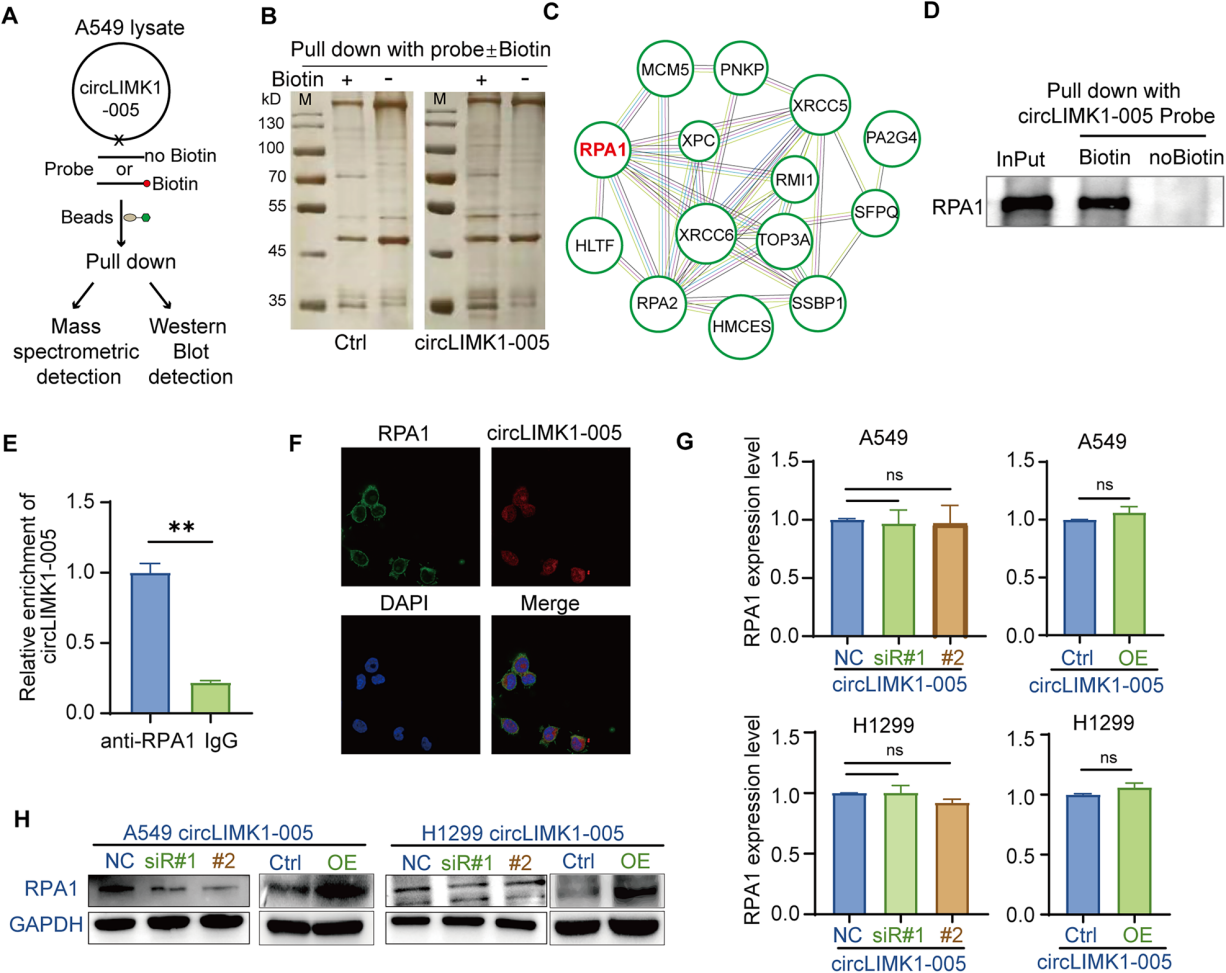
CircLIMK1 interacted with RPA1 and increased the protein level. **A**, **B** Specific biotin-labeled circLIMK1-005 probes were used to conduct RNA pull-down experiments in A549 cell lysates, followed by silver staining and mass spectrometry detection. **C** Protein-protein interaction network of the top proteins enriched with circLIMK1-005 biotin-labeled probe. **D** Pull-down assay and western blot with RPA1 antibody. **E** RIP with RPA1 antibody and RT-qPCR assay were used to detect enrichment of circLIMK1-005. **F** FISH with RPA1 antibody, circLIMK1-005 probe and DAPI in A549 cells. **G**, **H** RT-qPCR and western blot assay were conducted to detect relative expression of RPA1 regulated by circLIMK1-005 knockdown or overexpression in A549 and H1299 cells. **P value < 0.01.

To elucidate the molecular mechanism underpinning the activation of CDK4 signaling by circLIMK1-005, we aimed to identify factors that interacted with circLIMK1-005. Given that circLIMK1-005 was primarily localized in the cell nucleus (Fig. 1H, I), we postulated that its functional role might depend on protein interactions to trigger CDK4 signaling, as opposed to utilizing the conventional ceRNA mechanism. Thus, we performed an RNA pull-down assay using a biotinylated circLIMK1-005 probe, followed by silver staining and mass spectrometry analyses (Fig. 4A, B). Protein-protein interaction (PPI) network analysis of the top 30 candidate interacting proteins indicated that the network was largely composed of proteins related to DNA damage response, including RPA1, RPA2, HLTF, MCM5, XPC, and XRCC5/6 (Supplementary Table S3; Fig. 4C). Intriguingly, RPA1 emerged as the most significantly enriched protein in the biotinylated probe group compared to the control (Supplementary Table S2), as evidenced by mass spectrometry and corroborated by pronounced enrichment in the silver-stained gel at approximately 70 kDa (Fig. 4B). This led us to hypothesize that RPA1 could be a critical interaction partner of circLIMK1-005. To validate this hypothesis, we conducted an RNA pull-down experiment using a circLIMK1-005 probe, followed by a western blot assay. The data confirmed that RPA1 protein could be specifically captured by the biotinylated probe relative to the control probe (Fig. 4D). Subsequent RIP experiments with RPA1 antibody and RT-qPCR assay further revealed a significant enrichment of circLIMK1-005 in the anti-RPA1 immunoprecipitated samples compared to control IgG samples (Fig. 4E). FISH assays also substantiated the nuclear co-localization of circLIMK1 and RPA1 in A549 cells (Fig. 4F). Collectively, these results provided compelling evidence for the existence of a circLIMK1-005/RPA1 complex in LUAD cells.

We then focused on the effect of interaction on the RPA1 protein in A549 and H1299 cells. As determined by RT-qPCR, circLIMK1-005 knockdown and overexpression did not affect the expression of RPA1 mRNA (Fig. 4G). Interestingly, Western blot data demonstrated that the knockdown of circLIMK1-005 led to a significant reduction in RPA1 protein levels, whereas its overexpression resulted in a notable increase (Fig. 4H). These observations suggested that circLIMK1-005 exerted a post-transcriptional regulatory effect on RPA1 protein expression without affecting its mRNA levels. In summary, our findings uncovered that circLIMK1-005 interacted with and elevated the protein levels of RPA1, thereby implying a role for circLIMK1-005 in facilitating the progression of LUAD through the upregulation of RPA1 protein.

### RPA1 promoted the progression of LUAD and is involved in activating CDK4 signaling

**Fig. 5:**
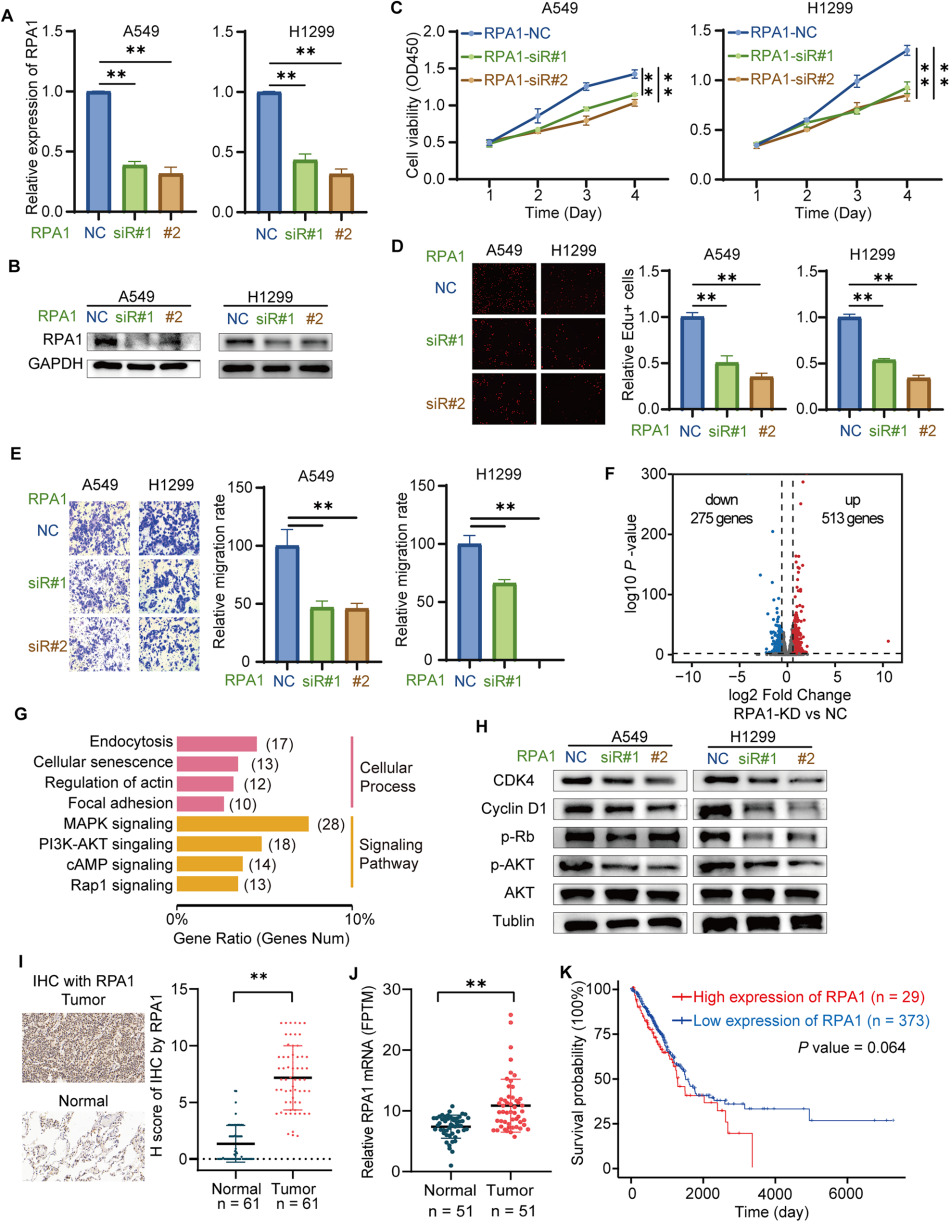
RPA1 facilitated the progression of LUAD and was involved in activating CDK4 signaling. **A**, **B** RT-qPCR and western blot assay were adopted to verify the efficiency of RPA1 knockdown. **C** CCK8 assay was adopted to detect cell viability. **D** EdU assay was used to detect the DNA replication rate. **E** Transwell assay was applied to assess the metastasis ability. **F** Volcano map of differentially expressed genes regulated by RPA1 using transcriptome sequencing. **G** KEGG analysis of differentially expressed genes. **H** Western blot with Cyclin D1, CDK4, RB, and AKT antibodies in RPA1-knockdown cells. **I** IHC with RPA1 antibodies and statistical analysis. **J** Relative expression level of RPA1 mRNA in NSCLC tissues and para-tumor normal tissues from ProteomeXchange database (IPX0001804000)**. K** Kaplan–Meier survival analysis of RPA1 in patients with LUAD. **P value < 0.01.

Although previous studies have hinted at the involvement of RPA1 in the pathogenesis of LUAD [19, 23, 24], its specific role and underlying mechanisms in disease progression have yet to be fully elucidated. To address this gap, we carried out functional studies on RPA1 using A549 and H1299 cells. Initially, we established RPA1-knockdown (RPA1 siR) cell lines in A549 and H1299 cells and verified the knockdown efficiency through RT-qPCR analysis (Fig. 5A, B). Further CCK8 and EdU assays demonstrated that the knockdown of RPA1 impaired cell proliferation in A549 and H1299 cells (Fig. 5C, D). Additionally, a transwell metastasis assay indicated that RPA1 knockdown significantly decreased the metastasis of A549 and H1299 cells (Fig. 5E). Collectively, RPA1 served as a key factor in promoting both the proliferation and metastasis of LUAD cells, thereby potentially contributing to LUAD progression.

The RPA complex, crucial for DNA replication, recombination, DNA damage repair [14–16], and transcriptional regulation [17–20], consists of several subunits, among which RPA1 is the largest and arguably the most functionally important. Given its significance, we sought to elucidate the regulatory mechanisms of RPA1 in the context of LUAD progression. Firstly, we conducted transcriptome sequencing for RPA1 KD A549 cells and NC. Transcriptomic analysis revealed 788 differentially expressed mRNAs, as visualized by a volcano plot (Fold change >1.5, *P* value < 0.05) (Fig. 5A). Subsequent KEGG pathway analysis of these differentially expressed genes highlighted endocytosis, cellular senescence, actin regulation, and focal adhesion as the top-affected cellular processes, along with MAPK, PI3K-AKT, cAMP, and RAP1 as the most significantly impacted signaling pathways (Fig. 5G). Interestingly, the enrichment pathway of RPA1 knockdown was consistent with that of circLIMK1-005 knockdown (Fig. 3B vs Fig. 5G). Given RPA1’s well-known involvement in DNA damage response and cell cycle regulation, we hypothesized that RPA1 might also modulate CDK4 signaling. To validate this, we performed western blot assays on RPA1-knockdown A549 and H1299 cells, probing for Cyclin D1, CDK4, phospho-Rb, and phospho-AKT. The assays revealed a significant reduction in levels of Cyclin D1, CDK4, phospho-Rb, and phospho-AKT upon RPA1 knockdown (Fig. 5H), indicating that RPA1 activated CDK4 signaling. Finally, the protein levels of RPA1 were detected using IHC in local LUAD patients. The IHC results confirmed that RPA1 proteins were highly expressed in tumor tissues compared to adjacent normal tissues (Fig. 5I). Additionally, we assessed the mRNA expression of RPA1 using the ProteomeXchange database (IPX0001804000) [40]. This analysis corroborated the protein data, revealing similar upregulation trends (Fig. 5I). Moreover, analysis on TCGA database showed that high RPA1 expression was probably correlated with poor prognosis in LUAD patients (Fig. 5k). Collectively, our findings underscored the pivotal role of RPA1 in promoting both cellular proliferation and metastasis in NSCLC while also activating CDK4 signaling pathways.

### CircLIMK1-005 promoted the progression of LUAD by regulating the RPA1/CDK4 axis

**Fig. 6:**
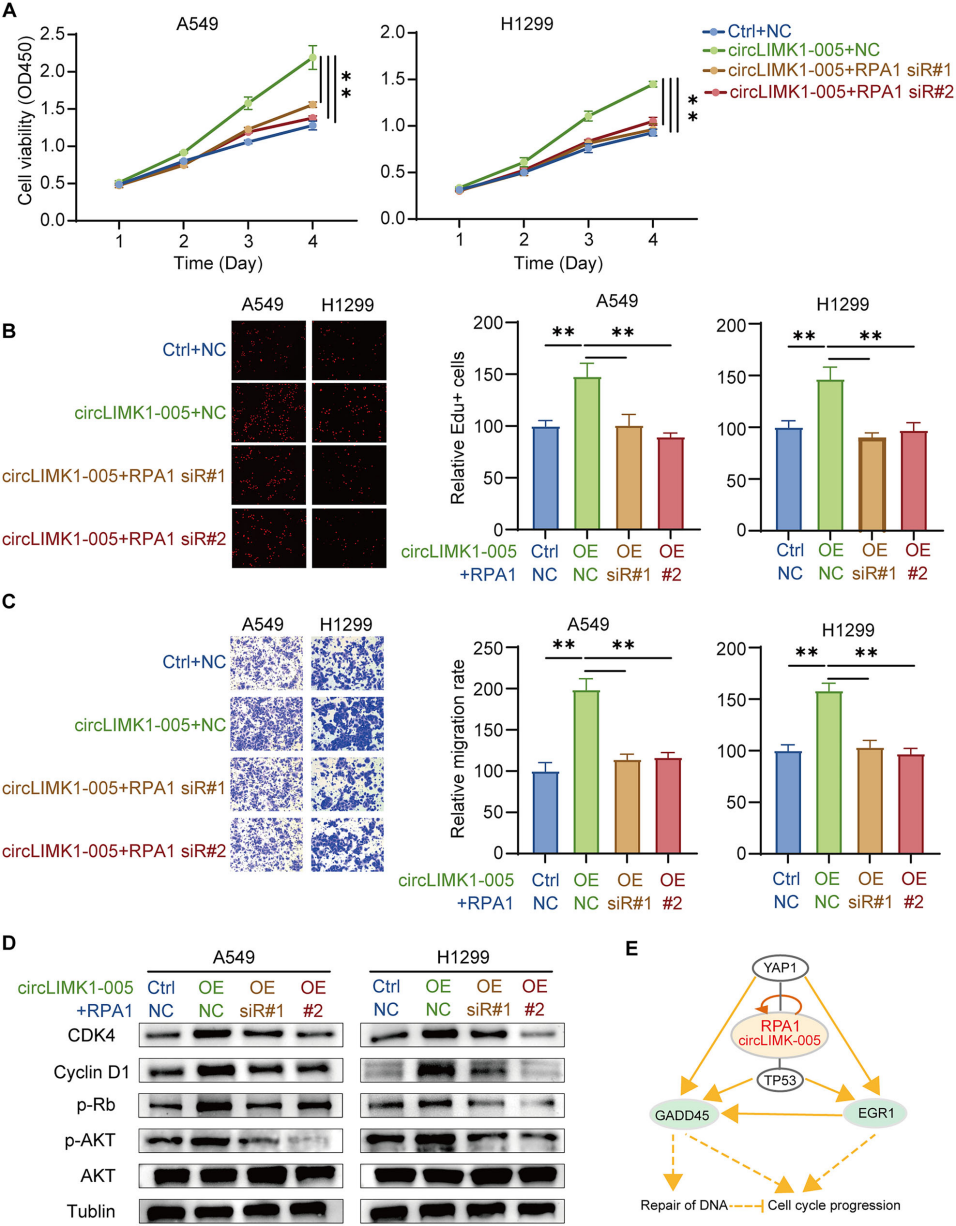
circLIMK1-005 promoted the progression of LUAD via regulating the RPA1/CDK4 axis. **A**–**C** CCK8, EdU and Transwell rescue experiments were implemented to confirm the role of RPA1 in the oncogenic function of circLIMK1-005. **D** Western blot was used to confirm the CDK4 signaling regulated by RPA1. **E** Ingenuity Pathway Analysis (IPA) analysis of the role played by circLIMK1-005 and RPA1 complex in DNA damage response and cell cycle progression. **P value < 0.01.

To further delineate the functional interplay between RPA1 and CDK4 within the context of circLIMK1-005 activity in A549 and H1299 cells, a series of functional rescue experiments were performed. Utilizing CCK8 and EdU assays, we observed that the proliferative enhancement triggered by circLIMK1-005 overexpression was substantially mitigated when RPA1 was concurrently knocked down (Fig. 6A, B). Similar results were observed in cell metastasis assays, underscoring the oncogenic role of RPA1 within the circLIMK1-005 framework (Fig. 6C). To substantiate these functional observations, we conducted western blot analyses probing for Cyclin D1, CDK4, phospho-RB, and phospho-AKT. The data revealed that elevations in these protein levels—induced by circLIMK1-005 overexpression—were attenuated upon RPA1 knockdown (Fig. 6D). These results further supported the involvement of RPA1 in the CDK4 signaling pathway mediated by circLIMK1-005. Incorporating transcriptomic databases for both circLIMK1-005 and RPA1, we undertook an integrative analysis of genes differentially regulated by both factors. PPI network analysis of these genes yielded three main functional clusters: cell cycle progression, classic AKT signaling, and extracellular matrix proteins (Supplementary Fig. S6). Further analysis via ingenuity pathway analysis suggested that the circLIMK1-005/RPA1 axis may influence DNA damage response and cell cycle progression through the upregulation of GADD45 and EGR1 genes, mediated by YAP1 and TP53 proteins (Fig. 6E). In summary, our collective findings proposed a mechanistic model wherein circLIMK1-005 advanced LUAD progression by potentiating the RPA1/CDK4 signaling axis. This work laid the groundwork for future studies aimed at elucidating the specific transcriptional mechanisms through which the circLIMK1-005/RPA1 complex modulated CDK4 signaling.

### The circLIMK1-005/RPA1/CDK4 axis in vivo promoted LUAD progression

**Fig. 7:**
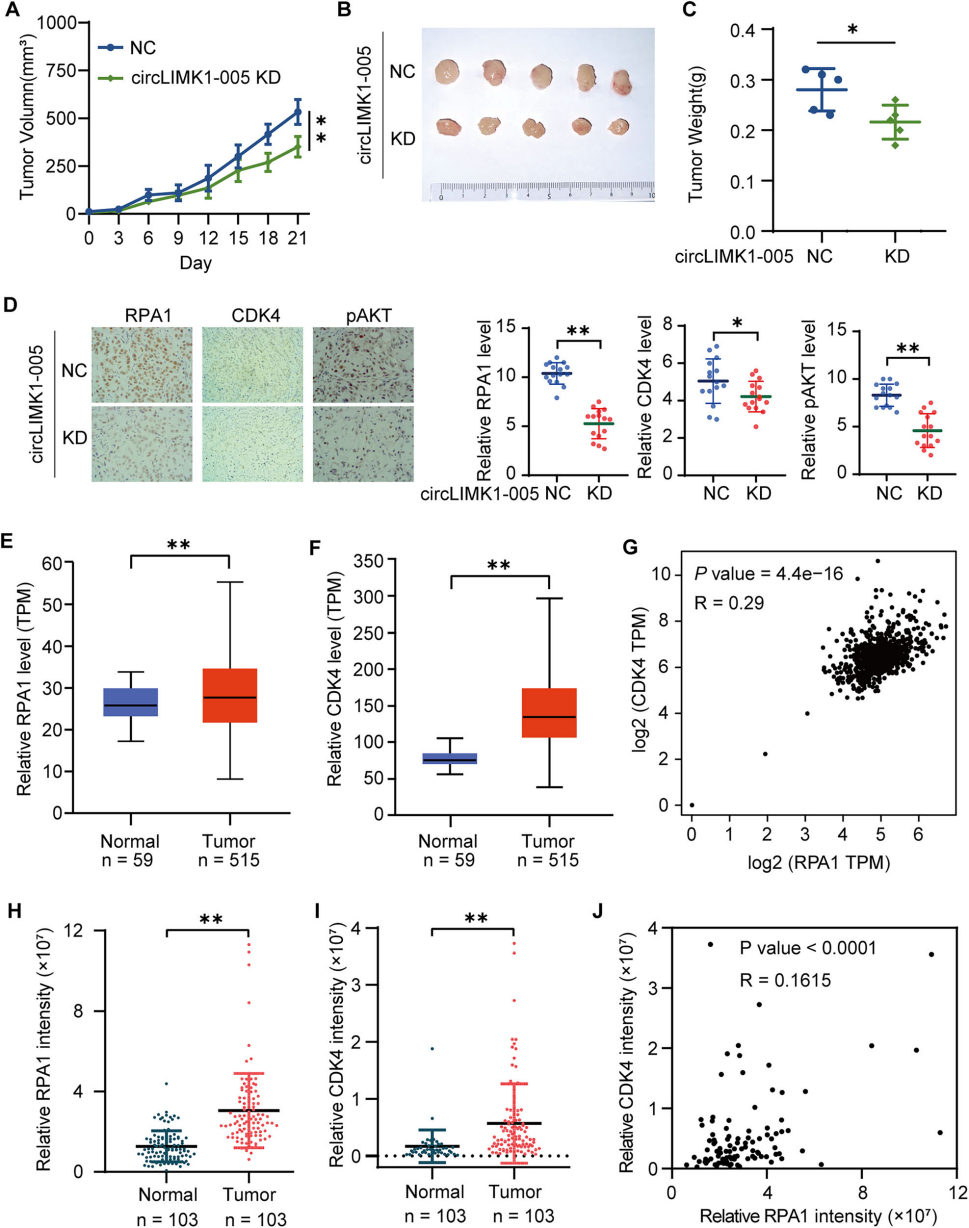
CircLIMK1-005/RPA1/CDK4 axis in vivo promoted LUAD progression. **A** Growth curves of A549 cell xenograft tumor. **B**, **C** A549 xenograft tumor and weight of the tumor. **D** IHC with RPA1 and CDK4 antibodies. **E**, **F** Relative expression of RPA1 and CDK4 mRNA in patients with LUAD from the TCGA database. **G** Correlation between RPA1 and CDK4 mRNA in patients with LUAD from the TCGA database. **H**, **I** Relative expression of RPA1 and CDK4 protein in patients with LUAD from ProteomeXchange database (IPX0001804000). **J** Correlation between RPA1 and CDK4 protein in patients with LUAD from ProteomeXchange database (IPX0001804000). *P value < 0.05, **P value < 0.01.

The significance of circLIMK1-005/RPA1/CDK4 in LUAD progression was investigated using the CDX model and clinical LUAD tissues. Initially, we established a xenograft model by injecting circLIMK1-005 knockdown A549 cells into the subcutaneous tissues of nude mice. Notably, the mice’s overall health was not impacted by the xenograft procedure (Supplementary Fig. S7). However, a marked suppression in tumor growth was observed in the circLIMK1-005 knockdown group, as substantiated by both tumor volume and weight measurements (Fig. 7A, C), thereby corroborating the pro-oncogenic role of circLIMK1-005 in LUAD in an in vivo context. Further, IHC using RPA1 and CDK4 antibodies revealed a decrease in the protein levels of RPA1 and CDK4 in the circLIMK1-005 knockdown group (Fig. 7D). These results highlighted the functional implication of the circLIMK1-005/RPA1/CDK4 axis in LUAD progression in vivo.

The correlation between RPA1 and CDK4 was analyzed in three LUAD cohorts. Firstly, the relative expression and association of RPA1 and CDK4 mRNA were assessed in patients with LUAD sourced from the TCGA database. Notably, both RPA1 and CDK4 mRNA levels were elevated in tumor tissues relative to adjacent normal tissues (Fig. 7E, F). Furthermore, a significant positive correlation was identified between RPA1 and CDK4 mRNA expression (Fig. 7G). Subsequently, we assessed the protein expression and correlation of RPA1 and CDK4 using the ProteomeXchange database (IPX0001804000) [40]. This analysis corroborated the mRNA data, revealing similar upregulation and correlation trends (Fig. 7H–J). Collectively, these data strongly supported the involvement of the RPA1/CDK4 axis in LUAD progression. In summary, our study elucidated the critical role of the circLIMK1-005/RPA1/CDK4 axis in the promotion of LUAD progression, both in vitro and in vivo.

## Discussion

A growing body of evidence underscores the pivotal role of circRNAs in the oncogenesis and progression of various cancers through diverse mechanisms [25, 26]. Intriguingly, circRNAs have been implicated in the dysregulation of their host genes and associated RNA-binding proteins in cancer cells [30], suggesting that the population of circRNAs exceeds that of canonical mRNA oncogenes in malignancies. In the present study, we identified 18 distinct isoforms of circLIMK1s (circLIMK1-001 to circLIMK1-018), all derived from the parent oncogene, LIMK1 (Supplementary Table S1). This diversity of isoforms amplifies the complexity of cancer pathogenesis. Despite the burgeoning interest in circRNAs as a substantive class of primarily non-coding RNAs, their translation into viable therapeutic targets remains a largely untapped area. Existing studies have highlighted the significance of over 20 circRNAs in LUAD [32, 33], yet the precise roles and underlying mechanisms of circRNAs in lung cancer remain predominantly enigmatic. Identifying the key oncogenic circRNAs and elucidating their associated pathways in LUAD represents a substantial scientific challenge. Our previous study performed sequencing on LUAD and adjacent normal tissues [34], identifying hundreds of differently expressed circRNAs. Notably, circHMGA2 (hsa_circ_0027446) was the first circRNA we investigated, revealing its role in metastasis and as a ceRNA in LUAD [34]. Meanwhile, we uncovered a novel circRNA, circLIMK1-005, markedly upregulated in LUAD patients’ tumor tissues. Accordingly, comprehensive analyses of circLIMK1-005 were performed to further delineate its role in LUAD progression.

In the present study, we have discovered a novel circular RNA, circLIMK1-005, which exhibited elevated expression in tumor tissues of LUAD patients. Notably, a positive correlation was found between elevated circLIMK1-005 expression and adverse prognostic outcomes in LUAD. Through in vitro experiments, we elucidated that circLIMK1-005 significantly enhances the proliferation and metastatic capabilities of LUAD cells. Mechanistically, we discovered that circLIMK1-005 acts as a potent activator of the CDK4 signaling pathway, thereby contributing to LUAD progression. Further investigation revealed circLIMK1-005 could interact with RPA1 protein (Fig. 4D, E), Our study shows that circLIMK1-005/RPA1 increases Cyclin D1 and CDK4 protein levels (Figs. 3G, 5H, and 6D) and regulates the mRNA expression of CCND1 and CDK4 (Fig. S6). Given the critical role of RPA complexes in genome stability and transcriptional regulation, we hypothesize that RPA1 contributes to the accumulation of Cyclin D1 and CDK4 by maintaining genomic stability or modulating transcription. Transcriptome analysis further suggests that circLIMK1-005/RPA1 regulates the AKT and MAPK pathways (Figs. 2B and 5G). Protein analysis confirms that circLIMK1-005/RPA1 promotes AKT phosphorylation (Figs. 3G, 5H, and 6D), supporting activation of the AKT pathway. These findings suggest that circLIMK1-005/RPA1 enhances Cyclin D1 and CDK4 accumulation via the AKT pathway. We propose that circLIMK1-005 binds to RPA1, stabilizing the RPA protein complex and activating the AKT pathway, which promotes Cyclin D1 and CDK4 accumulation and drives oncogenesis. Further studies are needed to elucidate the molecular mechanisms by which RPA1 regulates CDK4 protein accumulation.

In conclusion, our study highlighted the pivotal role of the circLIMK1-005/RPA1/CDK4 axis in the pathogenesis of LUAD. These findings suggested that circLIMK1-005 could serve as a promising therapeutic target for the clinical management of LUAD. Our research underscored the need for further in-depth studies to validate the potential of circLIMK1-005 as a novel therapeutic avenue. Next, we will continue to explore the mechanisms of post-transcriptional regulation of circLIMK1 and RPA1, as well as the mechanisms underlying the changes in RPA1 protein levels following the binding of circLIMK1 and RPA1. These are all issues that we need to address in the future. In addition, we believe that circRNAs can also reveal new functions in exploring the mechanisms of lung cancer development through single-cell level and spatial transcriptomic technologies.

## Materials and methods

### Clinical samples

Twelve patients with LUAD who underwent surgical resection at the Affiliated Cancer Hospital of Guangzhou Medical University (Guangzhou, China) were selected for tissue sample collection. Histopathological evaluation of the paraffin-embedded sections from these tissues was independently conducted by two certified pathologists. Importantly, none of the enrolled patients had undergone any preoperative treatments. Ethical approval for the use of these clinical specimens was granted by the Institutional Ethics Committee of the Affiliated Cancer Hospital of Guangzhou Medical University, and written informed consent was secured from all participating patients. In addition to these twelve patient samples, we obtained supplementary paraffin-embedded tissue sections, consisting of twenty matched pairs of LUAD and adjacent normal tissues, along with eighty-nine additional LUAD samples, from Servicebio (Wuhan, China).

### Cell culture

All human NSCLC cell lines PC9, HCC827, A549, H1299, and a human lung epithelial cell (bes-2b) were procured from the American Type Culture Collection (ATCC, USA). All cells were maintained in a controlled environment at 37 °C with 5% CO_2_ and in RPMMI 1640 medium (Biosharp, China). All media were supplemented with 10% (or 20% required) fetal bovine serum (FBS; Gibico, South America).

### RT-qPCR assay

Total RNA was isolated from cells employing SteadyPure Rapid RNA Extraction Kit (AG, China). cDNA was subsequently synthesized utilizing Evo M‐MLV RT Premix for qPCR AG11706 (AG, China). qPCR was performed with TB Green Premix Ex Taq II (Takara, China) with the following thermal cycling program: 95 °C for 30 s and 40 cycles at 95 °C for 5 s,60 °C for 30 s, and a dissociation step. The relative expression levels of target genes were computed using the 2^−ΔΔCt^ method. All primer sequences were synthesized (GeneCloudbio, China) as following:

GAPDH Forward: AATGGTGAAGGTCGGTGTGAACG, GAPDH Reverse: TCGCTCCTGGAAGATGGTGATGG; circLIMK1-005 Forward: ACCACCCCGAGTGTTTCATC, circLIMK1-005 Reverse: TGCAGTCACAACACCAGTACA; RPA1 Forward: CCAGTGCCCTATAATGAAGGA, RPA1 Reverse: CCATTCCCGAGCTTCCAT.

### Western blot assay

Cells were lysed using RIPA buffer supplemented with protease and phosphatase inhibitors (Biosharp, China). Following centrifugation, protein concentrations were quantified with a BCA Protein Assay Kit (Biosharp, China). Proteins were denatured at 100°C for 10 min using an odorless Dual-Color SDS-PAGE Loading Buffer (Beyotime, China). The denatured samples were resolved by 10% SDS-PAGE and electroblotted onto PVDF membranes (Millipore, USA). Membranes were blocked in 5% BSA (Biosharp, China) for 1 h at room temperature and then incubated overnight at 4 °C with primary monoclonal antibodies. Primary antibodies against human RPA1, TDP1, SMARCAL1, Cyclin D1, and GAPDH were sourced from Proteintech (China), while antibodies for AKT, phospho-AKT, Rb, and phospho-Rb were obtained from Abcam (USA). The CDK4 antibody was purchased from Cell Signaling Technology (USA). Following three 10-minute washes in TBST containing 0.1% Tween, membranes were incubated with secondary antibodies (Proteintech, China) for 1 h. Signal detection was performed using an ECL substrate (Biosharp, China).

### Actinomycin D RNA stability assay

A549 cells were treated with 5 mg/ml actinomycin D (Sigma, USA), and RNA samples were collected at 0, 4, 8, 12, and 24 h post treatment using the Zymo Quick RNA MiniPrep kit. RT-qPCR assays were conducted to measure RNA decay, normalizing the results to the 0-hour time point for each condition. The half-life of the RNA was calculated utilizing semi-logarithmic linear regression analysis to best fit the data.

### Cell transfection

To knock down circLIMK1-005 or RPA1 expression, specific small interfering RNAs (siRNAs) of circLIMK1-005(siR#1: CAAGCTGTACTGGTGTTGT; siR#2: CTGTACTGGTGTTGTGACT) and RPA1 (siR#1: GACCATTTGTGCTCGTGTT; siR#2: CGTGCTGTCTTCAAGCACTAT), respectively, along with control siRNA (NC) were designed and synthesized (GeneCloudBio, China). A549 and H1299 cells were transfected with these siRNAs utilizing Lipofectamine 3000 (Invitrogen, USA). Knockdown efficiency was validated 24 h post transfection through RT-qPCR and western blot assays. For stable knockdown, lentiviral vectors containing the target shRNAs were also synthesized (GeneCloudBio, China). Cells were infected with these lentiviruses in the presence of 1 μl Polybrene (5 μg/μl). After 72 h, puromycin selection (5 μg/ml) was initiated. Cells that became puromycin-resistant after 10 days were deemed stably transfected. To stably overexpress circLIMK1-005, a lentiviral vector carrying the full-length circLIMK1-005 gene was designed and synthesized (GeneCloudBio, China). The transduction and selection procedures were the same as those for stable knockdown. Either knockdown or overexpression efficiency was confirmed via RT-qPCR and western blot assays.

### LUAD cell-derived xenograft (CDX)

Male BALB/c nude mice (aged 4–5 weeks, SPF grade) were acquired from the Guangdong Experimental Animal Center (Guangzhou, China). The mice were randomly allocated into two groups, each consisting of 5 animals. Each mouse was subcutaneously implanted with 4 × 10^6^ A549 cells and housed under SPF conditions. Tumor dimensions in one of the groups were measured every three days, using the formula VT = (length × width^2^)/2 to calculate tumor volume. Ultimately, all mice were humanely euthanized, and their subcutaneous tumors were excised for subsequent analyses. All animal experiments were conducted in strict accordance with institutional animal care guidelines.

### Fluorescence in situ hybridization (FISH)

To measure the sub-cellular localization of circLIML1-005 in LUAD cells and to estimate the expression and location of circLIMK1-005 in both LUAD and adjacent normal tissues, a Cy3-labeled probe specifically targeting circLIMK1-005 (Probe sequence: CACTGCAGTCACAACACCAGTACAGCTTGGAGTG) was designed and synthesized (GeneCloudBio, China). The FISH assay was conducted using a commercial FISH kit (RiboBio, China). Briefly, A549 cells were trypsinized and resuspended in medium, and then approximately 4000 cells were seeded into a 48-well plate containing cover glass. Upon reaching 70–90% confluence, cells were washed thrice with PBS and fixed with 3.7% paraformaldehyde. Permeabilization was then carried out using 0.5% Triton-100 for 10 min at 4 °C. Cells were pre-hybridized at 37 °C for 30 min using a pre-hybridization buffer. Following this, the cells were incubated overnight at 37°C in the dark with a hybridization buffer containing the circLIMK1-005-specific FISH probe. After hybridization, cells were washed with graded concentrations of SSC solution and subsequently stained with DAPI for 15 min in the dark. For LUAD and paired adjacent normal tissues, paraffin sections were deparaffinized with xylene and rehydrated through a graded ethanol series, followed by proteinase K treatment for 5–10 min at 37 °C. The subsequent steps were the same as those carried out on A549 cells. Fluorescence imaging was finally achieved using a confocal microscope (Carl Zeiss AG, Jena, Germany).

### Cell proliferation assays

The Cell Counting Kit-8 (CCK8) assay was performed per the manufacturer’s guidelines (Biosharp, China). After the addition of the CCK8 reagent to each well, the 96-well plate was returned to the incubator for a 1-h incubation period. Subsequently, the optical density (OD) was measured for each well using a spectrophotometer.

For the EdU incorporation assay, cells in the 96-well plate were incubated with 10 μM EdU medium for 2 h. Following this, cells were treated with 100 μL of staining solution for 30 min. Nuclei were visualized using DAPI staining, and images were captured under a fluorescence microscope.

### Migration and invasion assays

For migration measurement, 20% FBS-supplemented RPMI 1640 medium was added to the lower chamber of a 24-well Transwell plate without Matrigel coating. Transfected cells were resuspended in serum-free medium and seeded into the upper chamber at a density of approximately 4 × 10^4^ cells per well in 200 μL of medium. After a 24-h incubation period, cells that had migrated from the upper to the lower chamber through the 8-μm pore membrane were fixed with 4% paraformaldehyde for 15 min. These cells were stained with 0.1% crystal violet for an additional 15 min. Finally, the migrated cells were counted under a microscope at ×20 magnification. In contrast to the migration experiment, the invasion assay required the addition of Matrigel (Corning, USA) to the upper chambers and an incubation time of 24 h. All other steps were consistent with those in the migration assay.

### RNA pull-down assay

To identify proteins interacting with circLIMK1-005, a biotin-labeled pull-down probe specifically targeting circLIMK1-005 and a control probe were designed and synthesized (GeneCloudBio, China). The RNA pull-down assay was performed utilizing the RNA pull-down kit (BersinBio, Bes5102). In brief, approximately 1 × 10^7^ A549 were lysed and sonicated for 30 min in a 4 °C water bath. Then, a 20-μL aliquot of the lysate was saved for RNA input, while an 80-μL aliquot was reserved for protein input. Subsequently, probes were added to the lysate and incubated at room temperature for 24 h, followed by the addition of 100 μL of streptavidin magnetic beads (MCE, USA) and further rotation for 2–4 h at room temperature. The magnetic beads were isolated utilizing a magnetic stand and washed five times with washing buffer supplemented with PMSF, Protease inhibitor, and RNase inhibitor. The beads were then resuspended in 1 mL of the same washing buffer; 100 μL was used for RNA purification, and the remaining 900 μL was used for protein extraction. For RNA extraction, the sample was treated with proteinase K (Sangon, China) and RNA PK buffer, incubated at 50 °C for 45 min, and then at 95 °C for 10 min to reverse cross-links. RNA was then isolated utilizing TRIzol reagent (Invitrogen, USA), reverse-transcribed to cDNA, and stored at −80 °C for subsequent analyses. For protein extraction, the remaining sample was mixed with 4× loading buffer and heated to 100 °C for 10 min. The supernatant was magnetically separated, and the proteins were subjected to mass spectrometry and Western blot analyses.

### RNA immunoprecipitation (RIP)

RIP experiments were carried out to validate the interaction between circLIMK1-005 and target proteins utilizing a BersinBio RNA Immunoprecipitation Kit (Bes5101). In brief, approximately 2 × 10^7^ A549 cells were harvested and lysed using RIP lysis buffer. The RNA complexes were then immunoprecipitated using an anti-RPA1 antibody (Abcam), with an anti-IgG antibody (Abcam) as a negative control. Following this, co-precipitated RNAs were extracted using TRIzol reagent (Invitrogen, USA) and subsequently quantified via RT-qPCR.

### Transcriptome sequencing

Total RNA was extracted from cells utilizing TRIzol reagent (Invitrogen, USA). RNA concentration and quality were assessed utilizing a NanoDrop ND-1000 Spectrophotometer (NanoDrop, USA) and an Agilent Bioanalyzer (Agilent, USA), respectively. Poly(A)-enriched RNA was isolated from 1 μg of total RNA through two rounds of purification using Oligo(dT) 25 magnetic beads (Thermo Fisher Scientific, USA). Subsequently, the purified RNA was fragmented using the Magnesium RNA Fragmentation Module (New England Biolabs, USA) at 94 °C for 5–7 min. The fragmented RNA samples were reversely transcribed into cDNA employing Superscript II Reverse Transcriptase (Invitrogen, USA) and were then sequenced utilizing an Illumina NovaSeq 6000 platform (ERB, China). Transcript levels were quantitatively assessed utilizing StringTie and edgeR software packages. Differentially expressed mRNAs and genes were identified utilizing a log2 (fold change) >1.5 or 2 and *P*-value < 0.05, as analyzed through the R package edgeR.

### Immunohistochemistry (IHC)

For IHC, paraffin-embedded sections of both LUAD and adjacent normal tissues were deparaffinized and rehydrated through xylene and graded ethanol series. The tissues were then subjected to IHC staining to evaluate the protein expression using antibodies against RPA1 (Abcam) and CDK4 (CST) at 4 °C overnight. Subsequently, the tissues were incubated with a secondary antibody (Servicebio, China) for 1 h at room temperature. Finally, the tissue sections were mounted using neutral resin and visualized under a fluorescence microscope (Olympus, Japan). Staining scores were assessed based on both area coverage and staining intensity. The percentage of the stained area was scored as follows: 0 for <5%, 1 for 5–25%, 2 for 25–50%, 3 for 50–75%, and 4 for >75%. Staining intensity was categorized as 0 for no staining, 1 for weak staining, 2 for moderate staining, and 3 for intense staining. The overall staining score was independently evaluated by two pathologists and calculated as the sum of the area and intensity scores. Samples with a total score of 6 or higher were considered to exhibit high expression, whereas those with a score below 6 were categorized as having low expression.

### Statistical analysis

Data analysis was performed using GraphPad Prism 8 software. Cell metastasis and protein expression were quantitatively assessed using ImageJ software. All in vitro experiments were carried out in triplicate, and the data are summarized as mean ± standard deviation. Statistical significance between the two experimental groups was determined using Student’s *t*-test via SPSS 20.0 software. OS rates were evaluated using the Kaplan–Meier method and analyzed by the log-rank test. A *p*-value of less than 0.05 was considered statistically significant.

## Supplementary information

Supplementary material

Supplementary material-Tables

## Data Availability

The data supporting the conclusion of this article are presented within the article and its additional files.

## References

[1] Siegel RL, Miller KD, Fuchs HE, Jemal A. Cancer statistics, 2022. CA Cancer J Clin. 2022;72:7–33. 35020204 10.3322/caac.21708

[2] Chen Z, Fillmore CM, Hammerman PS, Kim CF, Wong K. Non-small-cell lung cancers: a heterogeneous set of diseases. Nat Rev Cancer. 2014;14:535–46. 25056707 10.1038/nrc3775 PMC5712844

[3] The Cancer Genome Atlas Research Network. Comprehensive molecular profiling of lung adenocarcinoma. Nature. 2014;511:543–50. 10.1038/nature13385 PMC4231481 25079552

[4] Herbst RS, Morgensztern D, Boshoff C. The biology and management of non-small cell lung cancer. Nature. 2018;553:446–54. 29364287 10.1038/nature25183

[5] Hanahan D, Weinberg RA. Hallmarks of cancer: the next generation. Cell. 2011;144:646–74. 21376230 10.1016/j.cell.2011.02.013

[6] Asghar U, Witkiewicz AK, Turner NC, Knudsen ES. The history and future of targeting cyclin-dependent kinases in cancer therapy. Nat Rev Drug Discov. 2015;14:130–46. 25633797 10.1038/nrd4504 PMC4480421

[7] Goel S, Bergholz JS, Zhao JJ. Targeting CDK4 and CDK6 in cancer. Nat Rev Cancer. 2022;22:356–72. 35304604 10.1038/s41568-022-00456-3 PMC9149100

[8] Jamal-Hanjani M, Wilson GA, Mcgranahan N, Birkbak NJ, Watkins TBK, Veeriah S, et al. Tracking the evolution of non-small-cell lung cancer. N Engl J Med. 2017;376:2109–21. 28445112 10.1056/NEJMoa1616288

[9] Schrock AB, Frampton GM, Suh J, Chalmers ZR, Rosenzweig M, Erlich RL, et al. Characterization of 298 patients with lung cancer harboring MET Exon 14 skipping alterations. J Thorac Oncol. 2016;11:1493–502. 27343443 10.1016/j.jtho.2016.06.004

[10] Blakely CM, Watkins TBK, Wu W, Gini B, Chabon JJ, Mccoach CE, et al. Evolution and clinical impact of co-occurring genetic alterations in advanced-stage EGFR-mutant lung cancers. Nat Genet. 2017;49:1693–704. 29106415 10.1038/ng.3990 PMC5709185

[11] Fassl A, Geng Y, Sicinski P. CDK4 and CDK6 kinases: from basic science to cancer therapy. Science. 2022;375:eabc1495. 10.1126/science.abc1495 PMC9048628 35025636

[12] Patnaik A, Rosen LS, Tolaney SM, Tolcher AW, Goldman JW, Gandhi L, et al. Efficacy and safety of abemaciclib, an inhibitor of CDK4 and CDK6, for patients with breast cancer, non-small cell lung cancer, and other solid tumors. Cancer Discov. 2016;6:740–53. 27217383 10.1158/2159-8290.CD-16-0095

[13] Maréchal A, Zou L. RPA-coated single-stranded DNA as a platform for post-translational modifications in the DNA damage response. Cell Res. 2015;25:9–23. 25403473 10.1038/cr.2014.147 PMC4650586

[14] Bae SH, Bae KH, Kim JA, Seo YS. RPA governs endonuclease switching during processing of Okazaki fragments in eukaryotes. Nature. 2001;412:456–61. 11473323 10.1038/35086609

[15] Bhat KP, Cortez D. RPA and RAD51: fork reversal, fork protection, and genome stability. Nat Struct Mol Biol. 2018;25:446–53. 29807999 10.1038/s41594-018-0075-z PMC6006513

[16] Syed A, Tainer JA. The MRE11-RAD50-NBS1 complex conducts the orchestration of damage signaling and outcomes to stress in DNA replication and repair. Annu Rev Biochem. 2018;87:263–94. 29709199 10.1146/annurev-biochem-062917-012415 PMC6076887

[17] Core LJ, Waterfall JJ, Lis JT. Nascent RNA sequencing reveals widespread pausing and divergent initiation at human promoters. Science. 2008;322:1845–8. 19056941 10.1126/science.1162228 PMC2833333

[18] Fujimoto M, Takaki E, Takii R, Tan K, Prakasam R, Hayashida N, et al. RPA assists HSF1 access to nucleosomal DNA by recruiting histone chaperone FACT. Mol Cell. 2012;48:182–94. 22940245 10.1016/j.molcel.2012.07.026

[19] Liu P, Wu D, Duan J, Xiao H, Zhou Y, Zhao L, et al. NRF2 regulates the sensitivity of human NSCLC cells to cystine deprivation-induced ferroptosis via FOCAD-FAK signaling pathway. Redox Biol. 2020;37:101702. 32898818 10.1016/j.redox.2020.101702 PMC7486457

[20] Yin Q, Li Y, Zhou Z, Li X, Li M, Liu C, et al. RPA1 controls chromatin architecture and maintains lipid metabolic homeostasis. Cell Rep. 2022;40:111071. 35830798 10.1016/j.celrep.2022.111071

[21] Serrano MA, Li Z, Dangeti M, Musich PR, Patrick S, Roginskaya M, et al. DNA-PK, ATM and ATR collaboratively regulate p53-RPA interaction to facilitate homologous recombination DNA repair. Oncogene. 2013;32:2452–62. 22797063 10.1038/onc.2012.257 PMC3651755

[22] Wang G, Li Y, Wang P, Liang H, Cui M, Zhu M, et al. PTEN regulates RPA1 and protects DNA replication forks. Cell Res. 2015;25:1189–204. 26403191 10.1038/cr.2015.115 PMC4650420

[23] Wong L, Vizeacoumar FS, Vizeacoumar FJ, Chelico L. APOBEC1 cytosine deaminase activity on single-stranded DNA is suppressed by replication protein a. Nucleic Acids Res. 2021;49:322–39. 33330905 10.1093/nar/gkaa1201 PMC7797036

[24] Ni Z, Yao C, Zhu X, Gong C, Xu Z, Wang L, et al. Ailanthone inhibits non-small cell lung cancer cell growth through repressing DNA replication via downregulating RPA1. Br J Cancer. 2017;117:1621–30. 29024939 10.1038/bjc.2017.319 PMC5729430

[25] Kristensen LS, Jakobsen T, Hager H, Kjems J. The emerging roles of circRNAs in cancer and oncology. Nature reviews. Clin Oncol. 2022;19:188–206. 10.1038/s41571-021-00585-y 34912049

[26] Kristensen LS, Andersen MS, Stagsted LVW, Ebbesen KK, Hansen TB, Kjems J. The biogenesis, biology and characterization of circular RNAs. Nat Rev Genet. 2019;20:675–91. 31395983 10.1038/s41576-019-0158-7

[27] Zhong Y, Du Y, Yang X, Mo Y, Fan C, Xiong F, et al. Circular RNAs function as ceRNAs to regulate and control human cancer progression. Mol Cancer. 2018;17:79. 29626935 10.1186/s12943-018-0827-8 PMC5889847

[28] Zhou W, Cai Z, Liu J, Wang D, Ju H, Xu R. Circular RNA: metabolism, functions and interactions with proteins. Mol Cancer. 2020;19:172. 33317550 10.1186/s12943-020-01286-3 PMC7734744

[29] Wang S, Zhang K, Tan S, Xin J, Yuan Q, Xu H, et al. Circular RNAs in body fluids as cancer biomarkers: the new frontier of liquid biopsies. Mol Cancer. 2021;20:13. 33430880 10.1186/s12943-020-01298-z PMC7798340

[30] Zhang L, Gao J, Long X, Zhang P, Yang X, Zhu S, et al. The circular RNA circHMGB2 drives immunosuppression and anti-PD-1 resistance in lung adenocarcinomas and squamous cell carcinomas via the miR-181a-5p/CARM1 axis. Mol Cancer. 2022;21:110. 35525959 10.1186/s12943-022-01586-w PMC9077876

[31] Wang C, Tan S, Liu W, Lei Q, Qiao W, Wu Y, et al. RNA-Seq profiling of circular RNA in human lung adenocarcinoma and squamous cell carcinoma. Mol Cancer. 2019;18:134. 31484581 10.1186/s12943-019-1061-8 PMC6724331

[32] Wang C, Tan S, Li J, Liu W, Peng Y, Li W. CircRNAs in lung cancer - biogenesis, function and clinical implication. Cancer Lett. 2020;492:106–15. 32860847 10.1016/j.canlet.2020.08.013

[33] Li J, Zhang Q, Jiang D, Shao J, Li W, Wang C. CircRNAs in lung cancer- role and clinical application. Cancer Lett. 2022;544:215810. 35780929 10.1016/j.canlet.2022.215810

[34] Yu Z, Zhu X, Li Y, Liang M, Liu M, Liu Z, et al. Circ-HMGA2 (hsa_circ_0027446) promotes the metastasis and epithelial-mesenchymal transition of lung adenocarcinoma cells through the miR-1236-3p/ZEB1 axis. Cell Death Dis. 2021;12:313. 33762580 10.1038/s41419-021-03601-2 PMC7991034

[35] Zhang M, Tian J, Wang R, Song M, Zhao R, Chen H, et al. Dasatinib inhibits lung cancer cell growth and patient derived tumor growth in mice by targeting LIMK1. Front Cell Dev Biol. 2020;8:556532. 33344441 10.3389/fcell.2020.556532 PMC7746816

[36] Cai S, Ye Z, Wang X, Pan Y, Weng Y, Lao S, et al. Overexpression of P21-activated kinase 4 is associated with poor prognosis in non-small cell lung cancer and promotes migration and invasion. J Exp Clin Cancer Res. 2015;34:48. 25975262 10.1186/s13046-015-0165-2 PMC4443662

[37] Wang W, Mouneimne G, Sidani M, Wyckoff J, Chen X, Makris A, et al. The activity status of cofilin is directly related to invasion, intravasation, and metastasis of mammary tumors. J Cell Biol. 2006;173:395–404. 16651380 10.1083/jcb.200510115 PMC2063840

[38] Li Y, Li F, Wang Y, Song F, Qi L, Hu Q. Circ-LIMK1 regulates cisplatin resistance in lung adenocarcinoma by targeting miR-512-5p/HMGA1 axis. Open Med. 2022;17:1568–83. 10.1515/med-2022-0542 PMC9547352 36304135

[39] Gerstberger S, Jiang Q, Ganesh K. Metastasis. Cell. 2023;186:1564–79. 37059065 10.1016/j.cell.2023.03.003 PMC10511214

[40] Xu J, Zhang C, Wang X, Zhai L, Ma Y, Mao Y, et al. Integrative proteomic characterization of human lung adenocarcinoma. Cell. 2020;182:245–61. 32649877 10.1016/j.cell.2020.05.043

