# Supplementary material for: Circular RNA circLIMK1-005 promotes the progression of lung adenocarcinoma by interacting with RPA1 protein to activate CDK4 signaling
Source: Cell Death Discov. 2025 Jul 1;11:297. doi: 10.1038/s41420-025-02565-y (PMC12218172; doi:10.1038/s41420-025-02565-y)
Supplement: Supplementary file 1 — Supplementary material [file 41420_2025_2565_MOESM1_ESM.pdf]

Supplementary material

Xia Yang[1], Lu Liu[1], Zhongjian Yu[1], Yuanlin Chen[1], Shiting Xu[1], Meiyuan Liu[1] , Meng Wang[1],

Huili Guo[1] , Zhiwu Zhang[1], Bingjie Shan[1], Silin Cai[1], Mengting Pan[1], Jiangyu Zhang [1,*],

Fengpin Wang[1,*], Yanfang Zheng[1,*]

---

[1] Guangzhou Institute of Cancer Research, the Affiliated Cancer Hospital, Guangzhou Medical University 510095, Guangzhou, China.

Authorship note: Xia Yang, Lu Liu and Zhongjian Yu contributed equally to this work.

*Corresponding Author:

Yanfang Zheng, E-mail: zheng2020@gzhmu.edu.cn

Fengpin Wang, E-mail: 2022390018@gzhmu.edu.cn

Jiangyu Zhang, E-mail: zhangjiangyu@gzhmu.edu.cn

Guangzhou Institute of Cancer Research, the Affiliated Cancer Hospital, Guangzhou Medical University 510095, P.R. China.

Supplementary Tables

Table S1 Circular RNA cluster circLIMK1s generated from parent LIMK1 gene.

CircBank ID	Circbase ID	position	Length	Transcript
hsa_circLIMK1_001	hsa_circ_0080518	chr7:73498106-73511519	615	NM_002314
hsa_circLIMK1_002	hsa_circ_0080519	chr7:73498106-73522287	1366	NM_002314
hsa_circLIMK1_003	hsa_circ_0080520	chr7:73500077-73523366	1229	NM_002314
hsa_circLIMK1_004	hsa_circ_0080521	chr7:73500077-73536855	3113	NM_002314
hsa_circLIMK1_005	hsa_circ_0002690	chr7:73510951-73511519	249	NM_002314
hsa_circLIMK1_006	hsa_circ_0080522	chr7:73510951-73513568	456	NM_002314

hsa_circLIMK1_007	hsa_circ_0080523	chr7:73510951-73526037	1192	NM_002314
hsa_circLIMK1_008	hsa_circ_0080524	chr7:73510951-73526328	1258	NM_002314
hsa_circLIMK1_009	hsa_circ_0080525	chr7:73511409-73511519	110	NM_002314
hsa_circLIMK1_010	hsa_circ_0080526	chr7:73511409-73520573	590	NM_002314
hsa_circLIMK1_011	hsa_circ_0080527	chr7:73511409-73536855	2877	NM_002314
hsa_circLIMK1_012	hsa_circ_0134615	chr7:73520204-73520573	273	NM_002314
hsa_circLIMK1_013	hsa_circ_0080528	chr7:73520204-73534968	1015	NM_002314
hsa_circLIMK1_014	hsa_circ_0001715	chr7:73521339-73523366	403	NM_001204426
hsa_circLIMK1_015	hsa_circ_0080529	chr7:73522200-73534968	558	NM_002314
hsa_circLIMK1_016	hsa_circ_0080530	chr7:73525977-73526328	126	NM_002314
hsa_circLIMK1_017	hsa_circ_0080531	chr7:73526262-73530288	223	NM_002314
hsa_circLIMK1_018	hsa_circ_0001716	chr7:73535254-73535605	351	NM_001204426

Table S2 Full length sequence of CircLIMK1 (LIMK1 exo3, green; LIMK1 exo4, red)

GTGTTGTGACTGCAGTGCCTCCCTGTCGCACCAGTACTATGAGAAGGATGGGCAGCTCTTCTGCAAG
AAGGACTACTGGGCCCGCTATGGCGAGTCCTGCCATGGGTGCTCTGAGCAAATCACCAAGGGACTGG
TTATGGTGGCTGGGGAGCTGAAGTACCACCCCGAGTGTTTCATCTGCCTCACGTGTGGGACCTTTAT
CGGTGACGGGGACACCTACACGCTGGTGGAGCACTCCAAGCTGTACTG

Table S3 Candidates enriched by circLIMK1-005 pull down and mass spectrometr detection.

Candididates		circLIMK-005	Ctrl	circLIMK-005	Ctrl	Ratio
Accession	Protein	Bio_Probe	Bio_Probe	noBio_Probe	noBio_Probe	
P27694	RPA1	8650000000	4420000000	69800000	12000000	160
P15927	RPA2	2590000000	1100000000	3940000	1680000	657
P13010	XRCC5	237000000	49400000	9320000	9860000	15
P12956	XRCC6	235000000	49400000	10400000	6830000	17
P23246	SFPQ	201000000	17800000	11000000	8870000	11
P12268	IMDH2	122000000	84900000	663000	533000	173
P30101	PDIA3	76500000	1730000	5460000	814000	12
P11586	C1TC	52300000	16800000	1570000	1460000	23
P08865	RSSA	51800000	29500000	5160000	5530000	8
P04083	ANXA1	35500000	6970000	1330000	1280000	16
P27824	CALX	25400000	730000	420000	170000	44
Q14527	HLTF	1060000000	596000000	\	\	\
Q9NZC9	SMAL1	431000000	313000000	\	\	\
O95453	PARN	78400000	38100000	\	\	\
P54727	RD23B	69200000	56800000	\	\	\

Q13472	TOP3A	64500000	64500000	\	\	\
Q96FZ2	HMCES	62600000	36500000	\	\	\
Q9NUW8	TYDP1	48500000	31900000	\	\	\
Q01831	XPC	47700000	45600000	\	\	\
Q9H9A7	RMI1	36400000	23800000	\	\	\
Q04837	SSBP	32100000	7160000	\	\	\
Q02790	FKBP4	19000000	988000	\	\	\
P35237	SPB6	15100000	917000	\	\	\
Q96T60	PNKP	13000000	8460000	\	\	\
P33992	MCM5	12200000	274000	\	\	\
Q9UQ80	PA2G4	11900000	5480000	\	\	\
P53396	ACLY	11100000	528000	\	\	\
P23381	SYWC	10700000	745000	\	\	\

Supplementary Figures

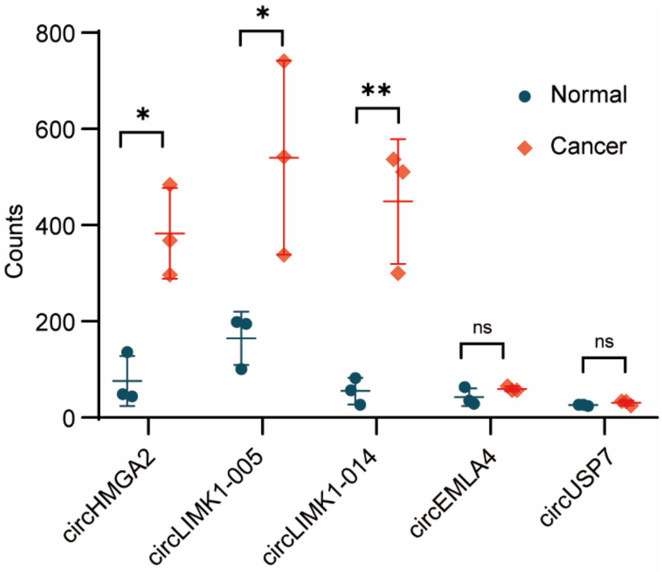

Fig. S1 Relative expression count of partial circRNAs, including circLIMK1-005 of interesting, and documented known circRNAs.

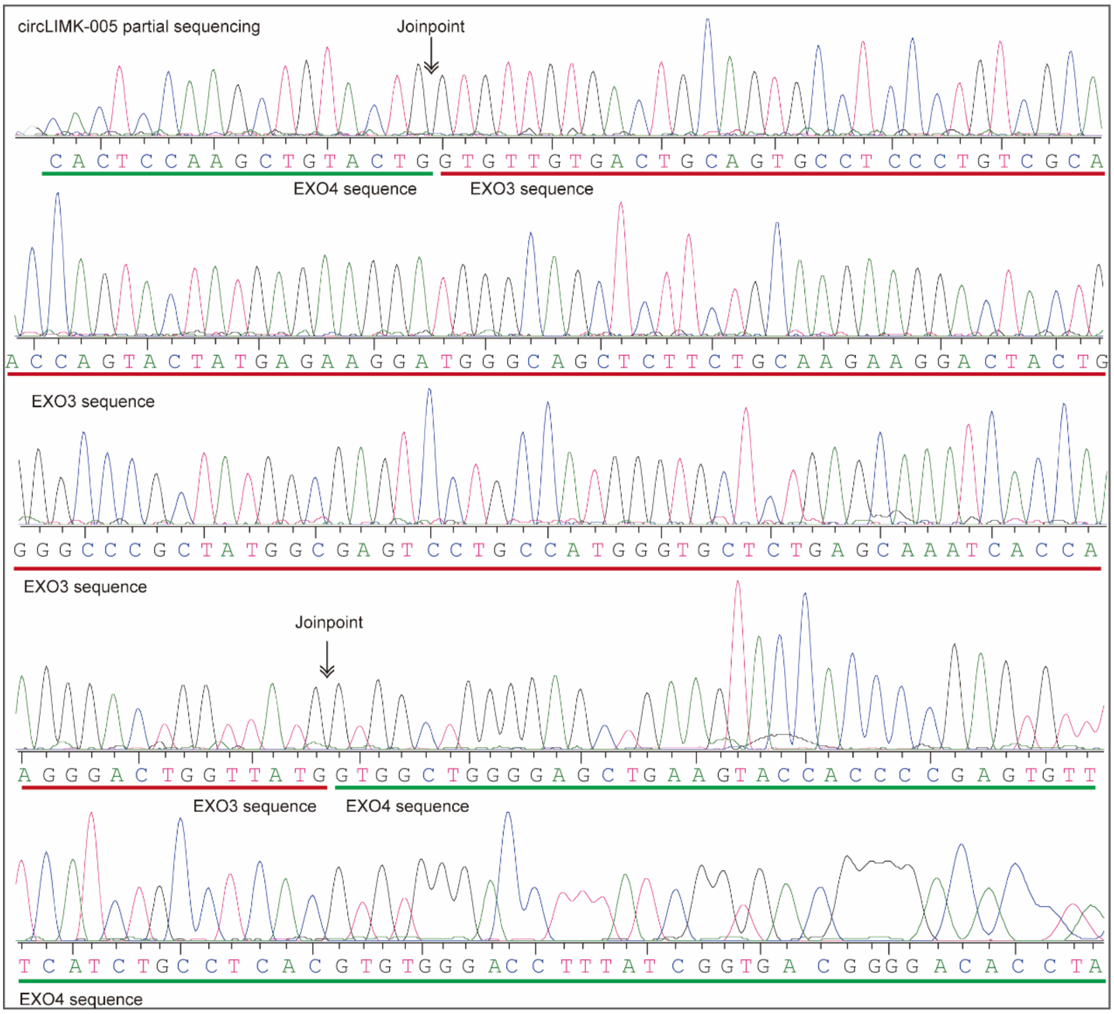

Fig. S2 Peak of sequencing on circLIMK1-005.

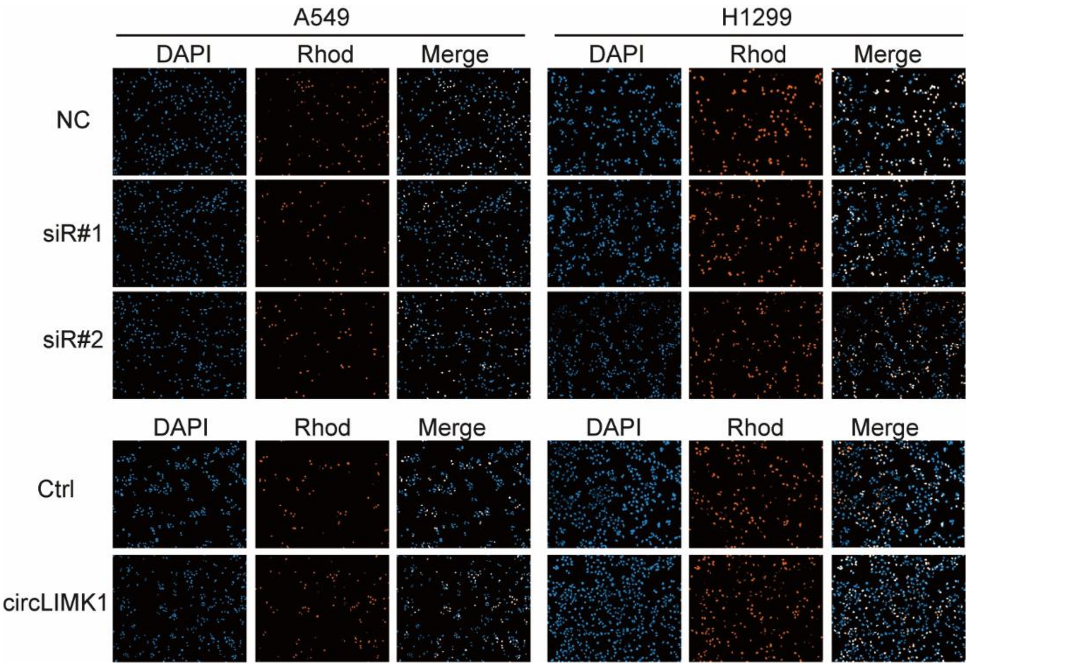

Fig. S3 EdU assay of circLIMK1-005 knockdown and overexpression in A549 and H1299 cells.

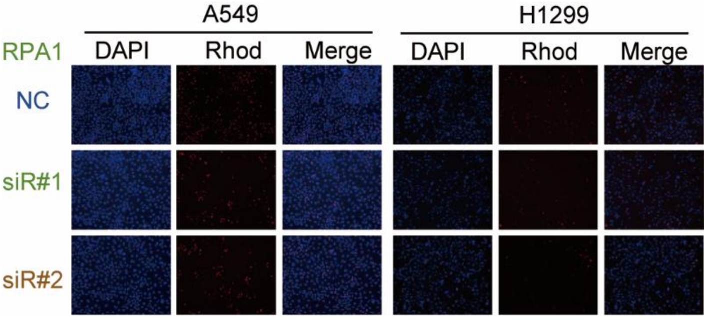

Fig. S4 EdU assay of RPA1 knockdown in A549 and H1299 cells.

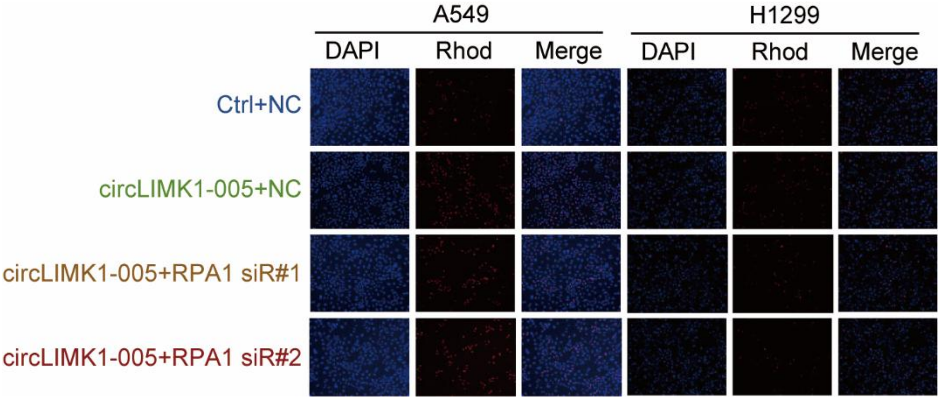

Fig. S5 EdU assay of circLIMK1-005 overexpression and RPA1 knockdown in A549 and H1299 cells.

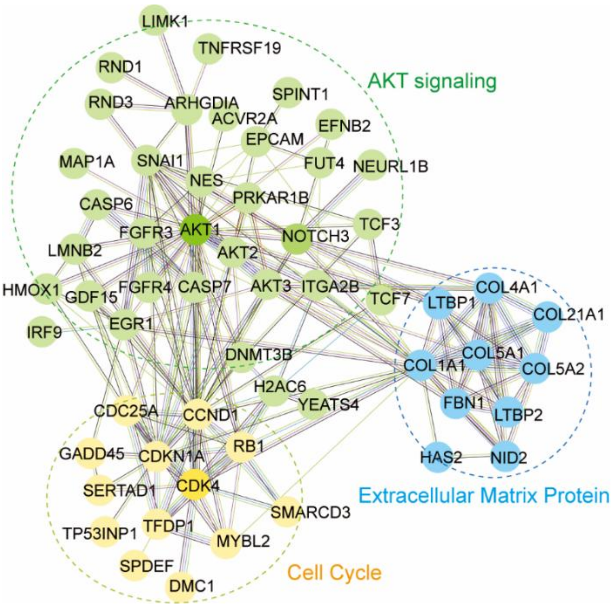

Fig. S6 Protein-protein interaction network of differentially expressed genes integrated from circLIMK1-005 and RPA1 knockdown transcriptome sequencing.

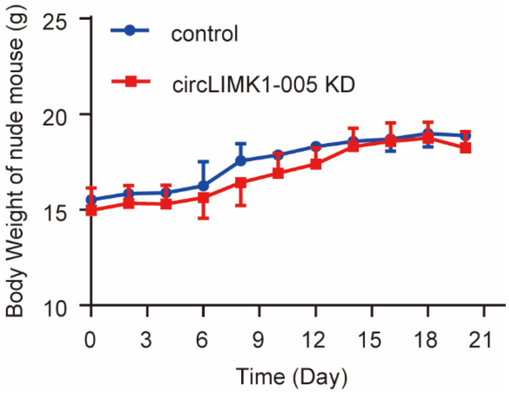

Fig. S7 Growth curves of nude mouse.
