# Supplementary material for: Circular RNA circLIMK1-005 promotes the progression of lung adenocarcinoma by interacting with RPA1 protein to activate CDK4 signaling
Source: Cell Death Discov. 2025 Jul 1;11:297. doi: 10.1038/s41420-025-02565-y (PMC12218172; doi:10.1038/s41420-025-02565-y)

Original western blots used in Fig3G

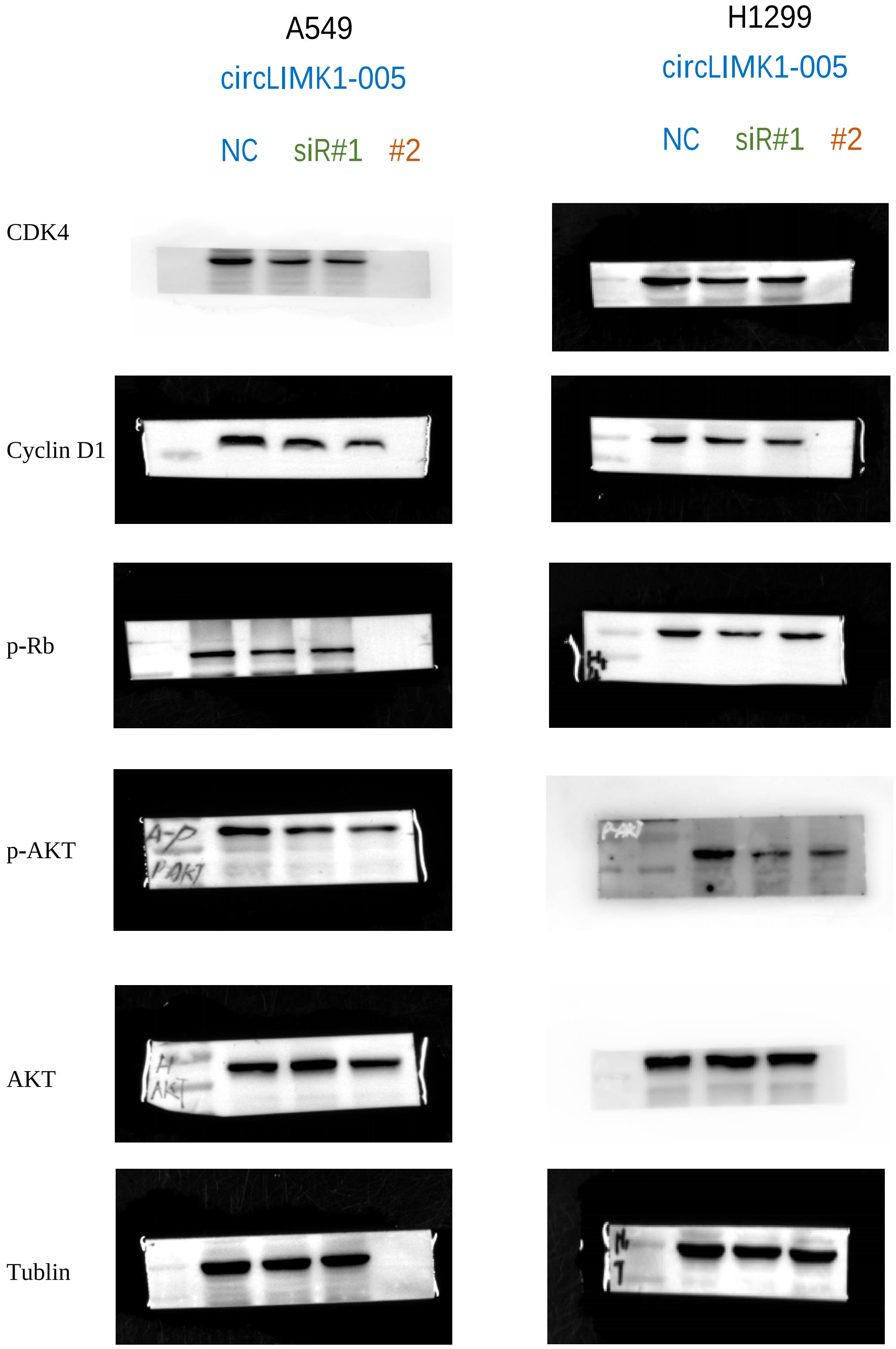

Original western blots used in Fig3G

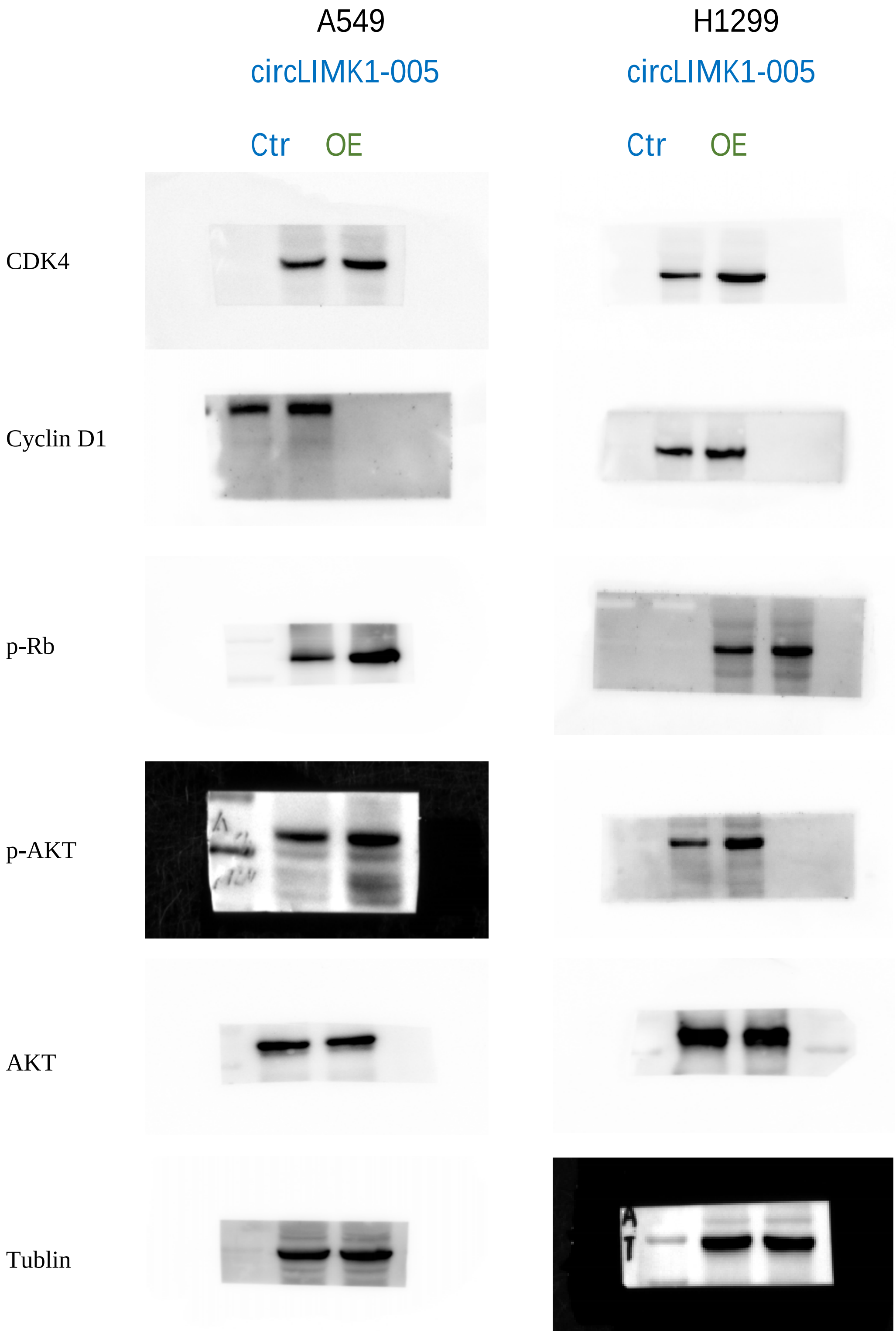

Original western blots used in Fig4D

Fig4D

Pull down with
circLIMK1-005 probe

Input Biotin noBiotin

RPA1

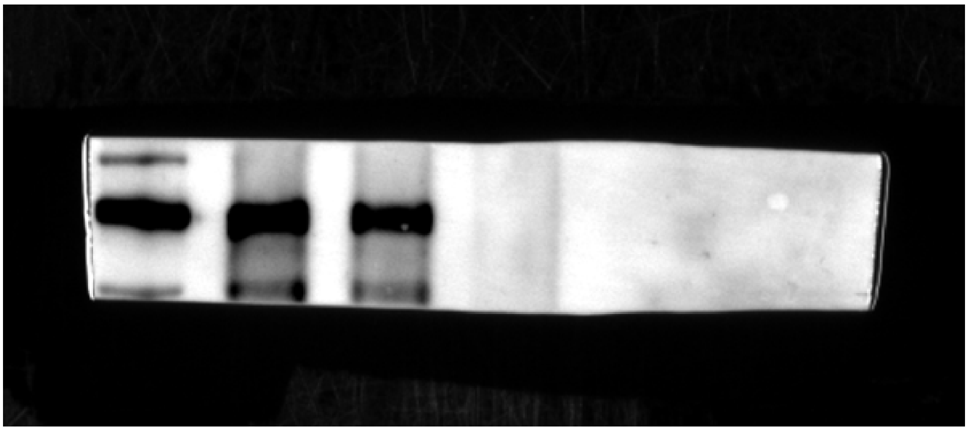

Original western blots used in Fig4H

A549

circLIMK1-005

NC siR#1 #2

RPA1

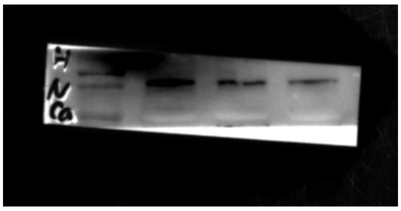

GAPDH

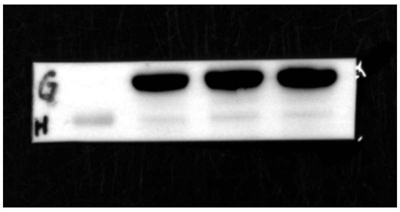

A549

circLIMK1-005

Ctr OE

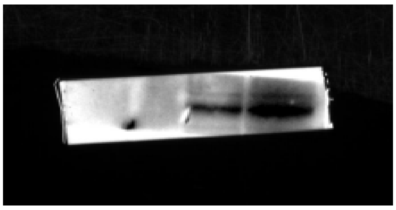

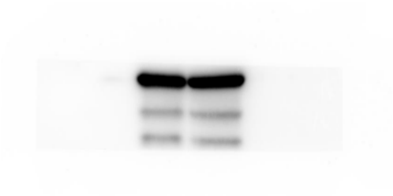

H1299

circLIMK1-005

NC siR#1 #2

RPA1

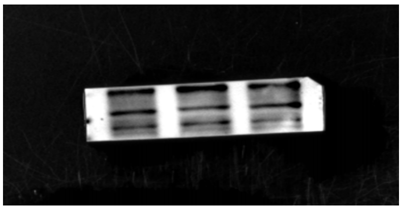

GAPDH

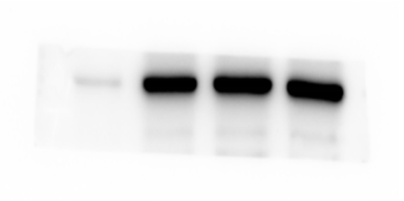

H1299

circLIMK1-005

Ctr OE

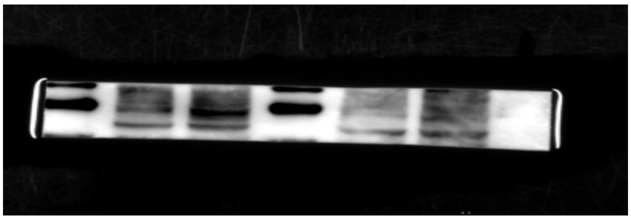

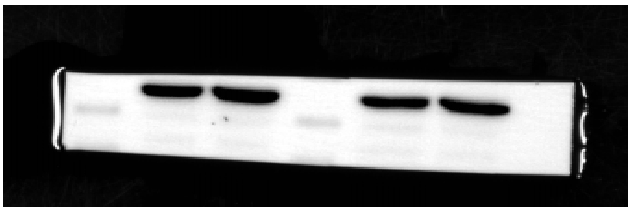

Original western blots used in Fig5D

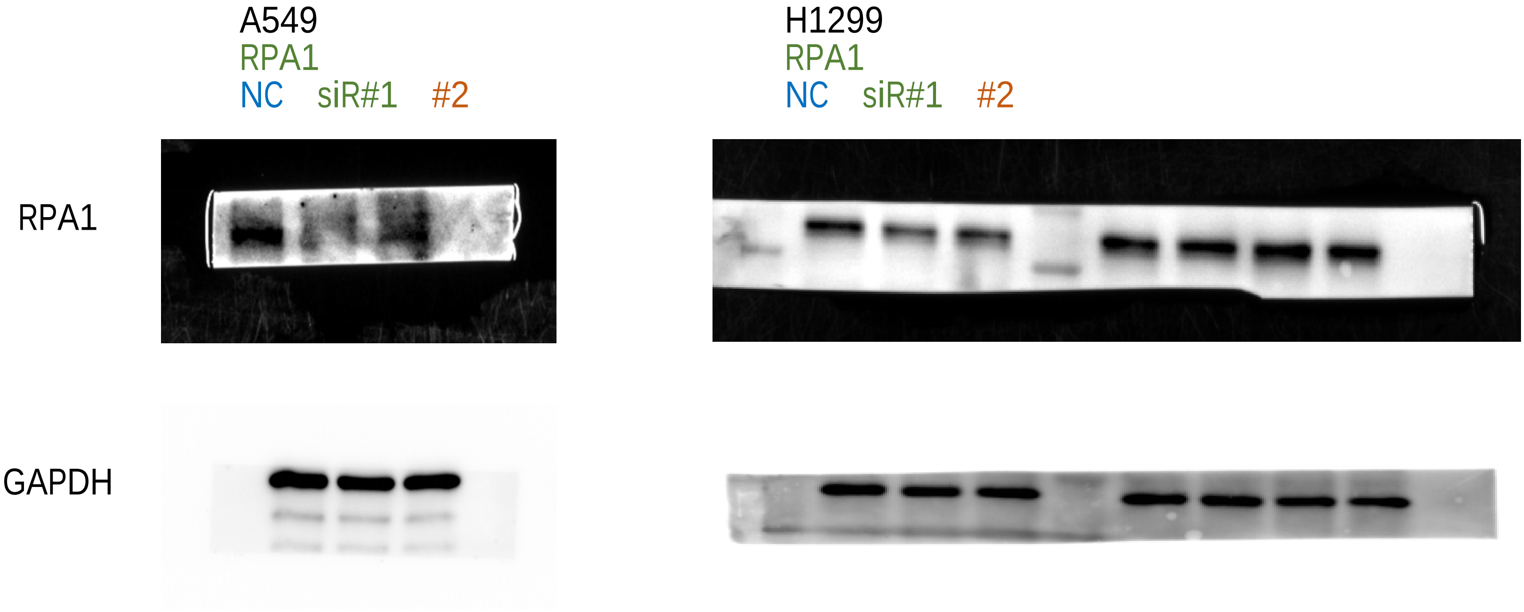

Original western blots used in Fig5H

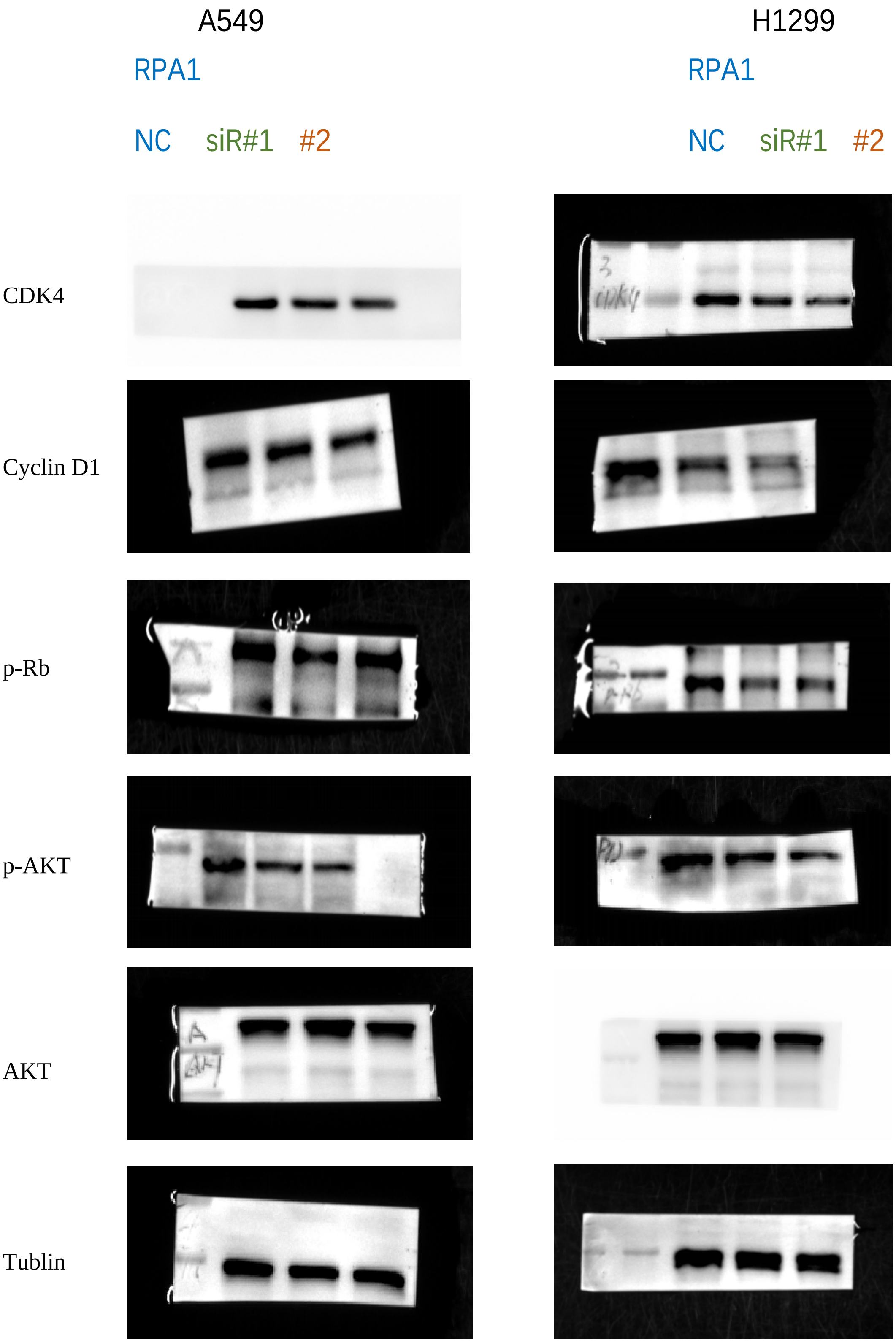

Original western blots used in Fig6D

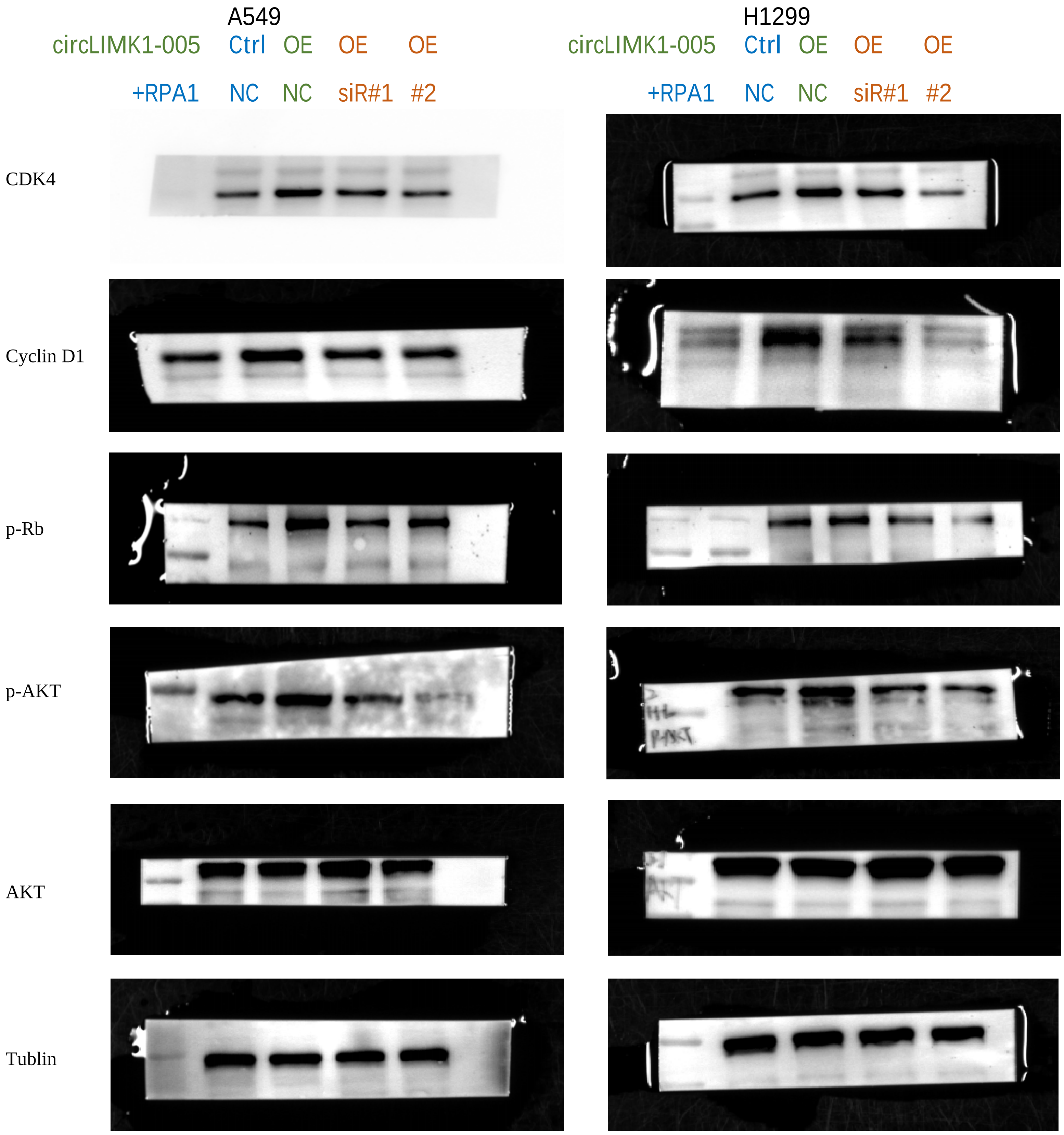

Supplement: Supplementary file 2 — Supplementary material-Tables [file 41420_2025_2565_MOESM2_ESM.pdf]
